# Hybrid ML and metaheuristic optimization of slag-fly ash-gypsum modified solidified sludge for construction

**DOI:** 10.1038/s41598-026-47428-3

**Published:** 2026-04-13

**Authors:** Hojatallah Azarkhosh, Yong Chen, Said Elias

**Affiliations:** 1 College of Civil Engineering and Architecture, Zhejiang University, Hangzhou, 310058 China; 2 Marie Skłodowska-Curie Actions (MSCA) Postdoctoral Fellow, Institute for Risk and Reliability, Leibniz University Hannover (LUH), Hannover, Germany; 3 Visiting Professor at Széchenyi István University, Administrative Building 103, University Square 1, Gyor-Moson-Sopron, Hungary

**Keywords:** Municipal sludge, Solidification, Optimization, Sustainable construction, Waste recycling, Engineering, Environmental sciences, Materials science

## Abstract

Conventional sludge disposal, including incineration and landfilling, is unsustainable and can cause secondary pollution; thus, sludge solidification is emerging as a sustainable alternative. This study aims to combine machine learning (ML) and metaheuristic optimization to maximize the unconfined compressive strength (UCS) of municipal sludge modified with slag, desulfurized gypsum, and fly ash. A total of 190 specimens were tested, and predictive models based on Gradient Boosting Machine (GBM), Random Forest (RF), Support Vector Regression (SVR), LightGBM, XGBoost, CatBoost, K-Nearest Neighbors (KNN), and Histogram Gradient Boosting (HistGBoost) were coupled with the Whale Optimization Algorithm (WOA). In addition, Particle Swarm Optimization (PSO), Genetic Algorithm (GA), Grey Wolf Optimizer (GWO), Gazelle optimization algorithm (GOA), Octopus Optimization Algorithm (OOA), Hiking Optimization Algorithm (HOA), and Young’s double-slit experiment optimizer (YDSE) were applied for comparison. Sensitivity analysis identified optimal WOA–ML parameter settings. The results demonstrated that the WOA–RF model outperformed all metaheuristic and other WOA–ML approaches by achieving the highest predicted UCS (8.29851 MPa). The WOA-ML models yielded an average optimal mix comprising sludge (44.2%), gypsum (19%), slag (18.7%), fly ash (16%), and NaOH (2.1%). Among the metaheuristic algorithms, PSO, GOA, OOA, TJO, DOA, GA, and YDSE demonstrated competitive performance. GWO achieved the highest UCS (8.226109 MPa), while HOA yielded the lowest (5.15366 MPa). The optimal mix averaged 38.9% sludge, 23.7% gypsum, 21.6% fly ash, 13.4% slag, and 2.5% NaOH. Partial dependence analysis confirmed the nonlinear effects of these parameters, while SHAP sensitivity analysis validated the optimization results. RSM validation further confirmed that both WOA–ML and metaheuristic approaches reliably predict the optimal UCS of modified sludge.

## Introduction

In recent years, with rapid urbanization worldwide, especially in China, wastewater treatment facilities have expanded significantly, leading to the production of large amounts of sludge. For example, China produced over 100 million tons of sludge in 2025^1^. Sludge is an inevitable byproduct of urban sewage treatment, and despite improvements in treatment capacity, the issue of prioritizing liquid effluent over solid waste management remains unresolved. Improper disposal poses serious environmental risks and hidden dangers, making standardized and safe treatment urgent. Consequently, the safe and stable disposal of sludge remains a major bottleneck restricting the sustainable development of the urban wastewater treatment sector^2,3^.

Conventional methods of sludge disposal, such as incineration and landfilling, often lead to secondary pollution and are increasingly unsustainable. An alternative and more sustainable approach is sludge solidification, which utilizes solidifying agents such as cement, fly ash, lime, and municipal solid waste incineration ash to stabilize sludge for use in construction materials, such as landfill cover layers. However, untreated sludge typically has high water content and low mechanical strength, making it unsuitable for direct application in construction. To enhance its suitability for use in construction, extensive research has been undertaken to reduce water content and increase the mechanical strength of solidified sludge. Recently, many scholars have applied machine learning (ML) to predict the unconfined compressive strength (UCS) of materials such as fly ash, concrete, and solidification materials^4–11^. ML models are commonly categorized into single and hybrid models^12^. Recent studies have applied DEM-driven approaches combined with AutoML to predict the macroscopic behavior of cementitious composites with variable frictional parameters^58,61^. Sharad Dadhich^13^ used a single model to forecast the UCS of concrete. Gil et al.^14^ applied various ML models, including XGBoost and Random Forest (RF), to predict the UCS of self-compacting concrete, with the RF model yielding the best results. Hemn Unis Ahmed et al.^15^ applied an SVR-GWO model for UCS prediction in geopolymer concrete. Feng et al.^16^ demonstrated that sulfoaluminate cement (SAC) combined with municipal solid waste incineration fly ash (MFA) can effectively solidify high-salt leachate sludge. The optimal MFA dosage enhanced unconfined compressive strength through ettringite and C–S–H gel formation, while heavy metals and PCDD/Fs were effectively immobilized^16^. Wang et al.^17^ investigated dredged sludge solidified with a novel GCP blend, showing that the combination of ettringite (AFt) and C-(A)-S–H gels significantly improved unconfined compressive strength and durability under wet-dry and freeze–thaw cycles compared to ordinary Portland cement. The study highlighted the microstructural mechanisms leading to a denser, more stable matrix for high-water-content sludge^17^. Zhu et al.^18^ investigated a low-carbon binder (CFS) for urban sludge stabilization, using Portland cement, fly ash, and steel slag. Their study showed improved compressive strength and reduced permeability, with microstructural analysis identifying ettringite, C-S–H gel, and calcite as key products, offering superior mechanical and environmental performance compared to Portland cement^18^. Yuan et al.^19^ developed a landfill cover material using lake sediment stabilized with fly ash, slag, desulfurization gypsum, and construction waste. The study showed improved mechanical properties and water retention, with microscopic analysis revealing the formation of ettringite, gypsum, and C-(A)-S–H gels, contributing to a stable matrix. Response surface methodology optimized material ratios, enhancing early strength and co-disposal of sludge and industrial waste^19^.

For sustainable structural solution, Liu et al.^20^ proposed recycled concrete–corrugated steel web girders, noting increased cracking with recycled aggregate. Permanoon et al.^21^ found MSPF enhances fracture toughness but requires optimal fiber content. Dong et al.^22^ showed basalt fibre reinforces recycled concrete for earthquake^23^ debris reuse. Khan et al. (2025) demonstrated rice husk ash can produce durable geopolymer bricks from agricultural waste. Wu et al.^24^ developed hybrid and spatiotemporal AI models for high arch dam deformation and safety assessment, improving monitoring while addressing structural vulnerabilities. Rezzoug et al.^25^ applied hybrid AI to predict flood energy reduction, supporting sustainable flood mitigation despite complex hydrodynamics. Recent advances in sustainable construction highlight both material innovation and structural performance. Li et al.^26^ reviewed multi-source solid waste–derived ceramic foams, highlighting their sustainability potential. Liu et al.^27^ developed nanoscale carbonated steel slag via MDEA-assisted carbonation, enhancing CO₂ sequestration while facing process and handling constraints. Tamoor and Zhang^28^ found slag and rice husk ash concretes balance environmental impact and cost, while foam concrete offers the lowest carbon footprint. Guo et al.^29^ developed low-carbon mortar from industrial wastes, and Wan et al.^30^ created low-fiber engineered geopolymer composites achieving high strength and ductility with reduced cement use. On structural behavior, Wang et al.^31^ showed that coupled traffic loading and dry–wet cycles degrade silty clay subgrades, while filter cake and slurry pressure mitigate stratum disturbance in shield tunneling. Lu et al.^32^ incorporated environmental factors into an elastoplastic concrete model, and Xu et al.^33^ showed that Fe₃O₄ enriched interlayers control matte aggregation in copper smelting, highlighting high-temperature microstructural challenges.

Wang et al.^34^ studied sludge solidification using red mud, coal gangue, and calcium carbide slag, achieving a 28-day UCS of 2.37 MPa with an optimal mix. Hydration products like C-S–H, C-A-H, and ettringite improved strength, while excess slag or moisture reduced performance^34^. Lei et al.^35^ evaluated sludge solidification with an ionic soil stabilizer and vacuum preloading. The method improved mechanical integrity, water stability, and heavy metal immobilization, offering a sustainable alternative to conventional cement-based treatment^35^. Yuan et al.^36^ enhanced dredged sludge using a novel curing agent (PB-NST) with a 1:1 mix of OPC and GGBFS, along with additives. The optimized mix achieved 4.75 MPa UCS at 7 days, 3.2 times higher than OPC alone^36^. Shi et al.^37^ studied the durability of sludge solidified with calcium carbide residue, desulfurization gypsum, and blast furnace slag under dry–wet cycles and sulfate erosion. UCS tests and microstructural analysis revealed strong resistance to both, with initial strength gains followed by moderate degradation. At higher sulfate concentrations, UCS recovered to 2.10 MPa^37^. Wang et al.^38^ developed a low-carbon ternary cementitious binder (LC3) for electroplating sludge stabilization, achieving 81.3% Cr and 97.8% Cd immobilization. This performance was attributed to the chemical binding of heavy metals in aluminate phases and C-A-S–H gels, enhanced by additional hydration products from the pozzolanic reaction^38^. Wang et al.^11^ developed an industrial waste-based binder (IWCB) for sludge solidification, combining slag, gypsum, cement, and bentonite. IWCB outperformed Portland cement, with 10% bentonite enhancing strength and reducing leaching. Liu et al.^39^ found that sodium leaching from C-(N-)A-S–H gels leads to partial decalcification, enhancing polymerization and water resistance. The study highlights the reduced durability of alkali-activated C-(N-)A-S–H gels compared to conventional C-(A-)S–H gels. Bashir et al.^40^ developed advanced machine intelligence (MI) models to predict the compressive strength of concrete incorporating fly ash and blast furnace slag as supplementary cementitious materials^40^. Noori Sichani et al.^15,41^ used machine learning and metaheuristic optimization to predict the compressive strength of fly ash-based geopolymer concrete^41^. Parhi et al.^42^ used machine learning and metaheuristic optimization to predict the mechanical strength of self-compacting alkali-activated slag concrete. Their findings suggest that similar methods could optimize sludge-based binders, improving both strength and sustainability in sludge solidification. Song et al.^17,43^ developed a method to recycle municipal solid waste incineration fly ash (IFA) and sewage sludge ash (ISSA) into low-carbon binders. The binder achieved over 40 MPa compressive strength, with chlorides immobilized by aluminate formation, demonstrating a sustainable approach for improving sludge-based construction materials^43^. Arachchilage et al.^18,44^ used machine learning to predict the UCS of alkali-activated slag-based cemented paste backfill. Their ensemble methods (gradient boosting regression, random forest) outperformed single models, with curing time and water-to-binder ratio being key factors, highlighting the potential of machine learning for optimizing slag-based binders^44^. Wang et al.^45^ explored using granulated blast furnace slag and flue gas desulfurization gypsum to solidify fly ash into cement-free backfill material. They found that 30 wt% fly ash improved strength, fluidity, and rheological properties, while reducing setting time and heavy metal leaching, showing potential for sustainable mine restoration and waste disposal^45^. Sludge is rich in organic matter, nitrogen, phosphorus, and potassium, offering substantial potential for resource recovery and low-carbon applications. However, the presence of toxic pollutants limits direct land application and necessitates robust treatment strategies that are both safe and environmentally sustainable. Using industrial solid wastes such as slag, desulfurization gypsum, and fly ash to modify and solidify municipal sludge, and subsequently applying the solidified materials as landfill cover layers, can reduce pollution from both industrial wastes and sludge, while decreasing dependence on natural clay. Discrete element modeling has been applied to polymer-modified asphalt mixtures to characterize microstructural behavior and bond interactions^59^, while industrial by-products such as sugarcane bagasse ash have been shown to enhance the mechanical performance and sustainability of hot-mix asphalt^60^. Furthermore, sustainability frameworks for recycled aggregate concrete produced with supplementary cementitious materials have provided guidance for eco-friendly binder design and circular construction practices^62^. In summary, relevant studies addressing this topic are scattered, and research in this area remains inadequate.

**Fig. 1:**
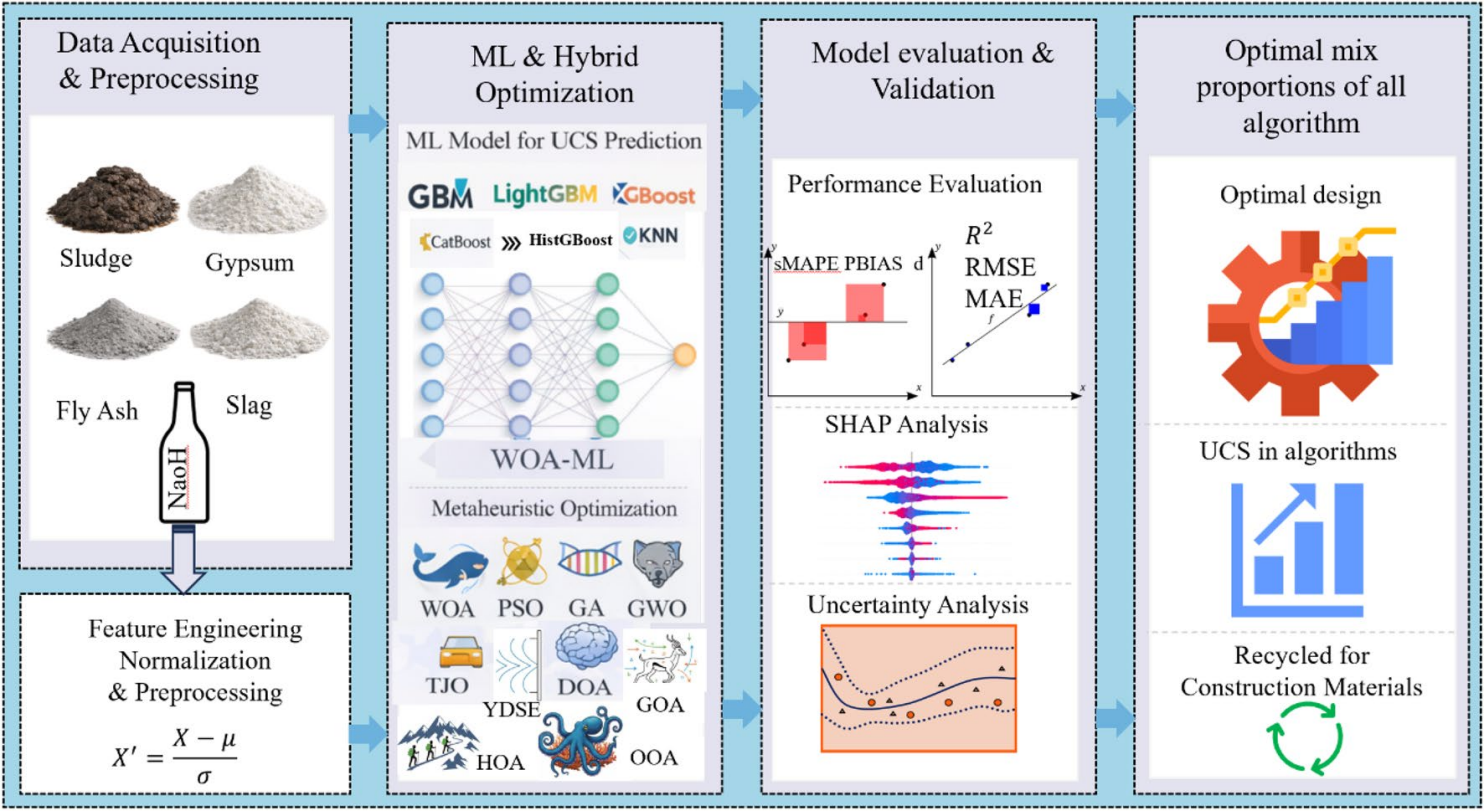
Overall workflow of the research.

Addressing this gap, the present study investigates the optimization of sludge solidification using slag, fly ash, gypsum blends for recycled sludge construction materials. Figure 1 shows the overall workflow. By integrating machine learning and metaheuristic-based optimization techniques, this research aims to predict and enhance the mechanical performance of sludge-based composites, providing a data-driven framework for sustainable material design. Specifically, hybrid WOA-ML models (such as GBM, LightGBM, XGBoost, CatBoost, KNN, HistGBoost, RF and SVR) were used for UCS prediction and optimization. The primary contribution of this study, in terms of algorithm fusion, is the development of an enhanced hybrid optimization algorithm and the identification of optimal parameters for sludge solidification. WOA was selected due to its demonstrated competitiveness among metaheuristic algorithms in optimizing complex neural networks; therefore, its enhancement directly improves the efficiency and effectiveness of the overall optimization framework. The WOA–ML hybrid’s exploration and exploitation capabilities allow it to avoid local optima and efficiently identify the global optimum. Furthermore, this framework was evaluated using various metaheuristic algorithm including WOA^46^, PSO^47,48^, GA^49^, GWO^50^, TJO^51^, DOA^52^, GOA^53^ OOA^54^, HOA^55^, and YSDE^56^ with a comprehensive performance evaluation across all models. Model verification was conducted by assessing the predictive accuracy of the models through standard validation methods, including error metrics and statistical tests. Sensitivity analysis was performed to evaluate the influence of key parameters, while uncertainty quantification (UQ) analysis was applied to ensure model robustness and reliability. Finally, the optimized results obtained from the WOA-ML and metaheuristic framework were validated through experimental simulations using Design Expert software. This approach not only addresses the technical bottleneck of sludge disposal but also advances circular-economy principles by promoting the reuse of urban waste in construction applications.

## Materials and methods

### Materials and characterization

**Fig. 2:**
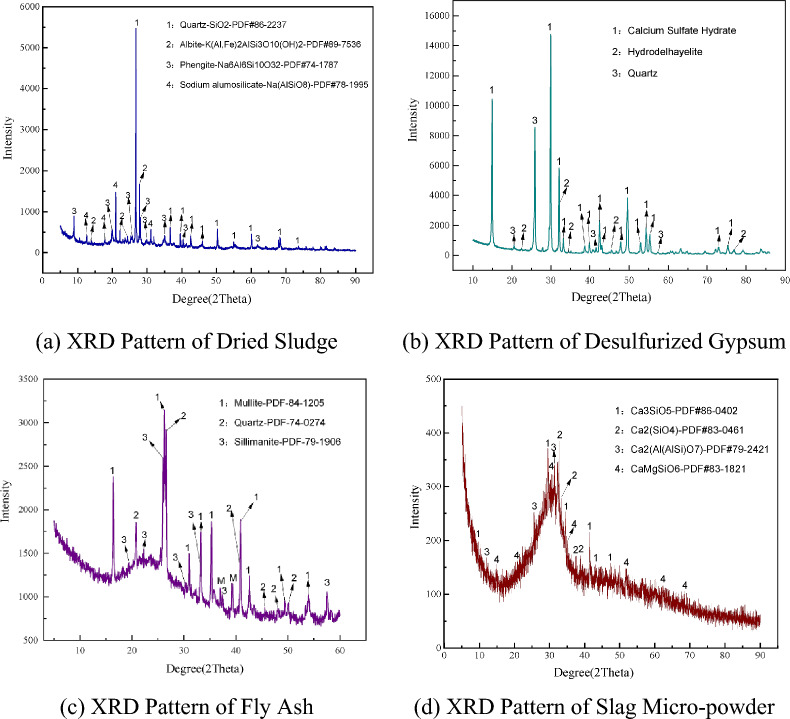
XRD Patterns and chemical composition of the materials^57^.

To investigate the solidification of municipal sewage sludge^57^the study includes dehydrated sludge, desulfurized gypsum, fly ash and slag all sourced from industrial facilities in China. The municipal sewage sludge, obtained from a wastewater treatment plant, is characterized by a deep brown color and a high moisture content of 75–80% on a wet basis. As detailed in Table 1, X-ray fluorescence (XRF) analysis indicates that the sludge is primarily composed of SiO₂ (39.46%), Al₂O₃ (11.10%), Fe₂O₃ (7.00%), and CaO (3.96%). Its mineralogical profile, shown in the X-ray diffraction (XRD) pattern in Fig. 2(a), is dominated by quartz (SiO₂), sodium feldspar (K(Al,Fe)₂AlSi₃O₁₀(OH)₂), phengite (Na₆Al₆Si₁₀O₃₂), and minor sodium alumosilicate (Na(AlSiO₈)). The desulfurized gypsum, sourced from a steel plant, appears as a white powder. Table 1 shows the XRF analysis of the gypsum, indicating that it is primarily composed of SiO₂ (31.89%), Al₂O₃ (12.45%), Fe₂O₃ (0.56%), and CaO (31.37%). Figure 2(b) presents the XRD pattern, which reveals that the mineral composition of the gypsum mainly consists of hydrated calcium sulfate, hydrodelhayelite, and quartz. The fly ash used in the experiment was obtained from a coal-fired power plant and appears as a gray powder. As listed in Table 1, the chemical composition of the fly ash, determined through XRF analysis, includes SiO₂ (47.17%), Al₂O₃ (27.15%), Fe₂O₃ (4.89%), and CaO (3.54%), classifying it as Class F fly ash. Figure 2(c) shows that XRD analysis reveals the mineral components of the fly ash primarily consist of quartz (SiO₂), mullite (Al₂(Al₂.₈Si₁.₂)O₉.₅₄), and sillimanite (Al₂SiO₅). Slag obtained from a steel plant is a grayish-white powder. As shown in Table 1, XRF analysis of the slag indicates that it contains SiO₂ (29.73%), Al₂O₃ (13.58%), Fe₂O₃ (1.01%), CaO (36.39%), MgO (6.56%), K₂O (0.55%), and Na₂O (0.28%). Figure 2(d) shows the XRD pattern, revealing that the primary mineral phases in the slag are hexagonal calcium silicate (Ca₃SiO₅), triclinic calcium silicate (Ca₂SiO₄), gehlenite (Ca₂Al(AlSi)O₇), and diopside (CaMgSiO₆).

**Table 1:** Chemical composition and content of materials.

Raw material	Chemical composition (%)							
	SiO_**₂**_	Al_**₂**_O_**₃**_	Fe_**₂**_O_**₃**_	CaO	MgO	K_**₂**_O	Na_**₂**_O	Others
Sludge	39.46	11.10	7.00	3.96	1.80	2.36	0.70	29.40
Desulfurized Gypsum	31.89	12.45	0.56	31.37	7.61	0.59	0.51	13.58
Fly Ash	47.17	27.15	4.89	3.54	0.378	1.391	0.68	13.70
Slag	29.73	13.58	1.01	36.39	6.56	0.55	0.28	42.30

### Experimental data collection

**Fig. 3:**
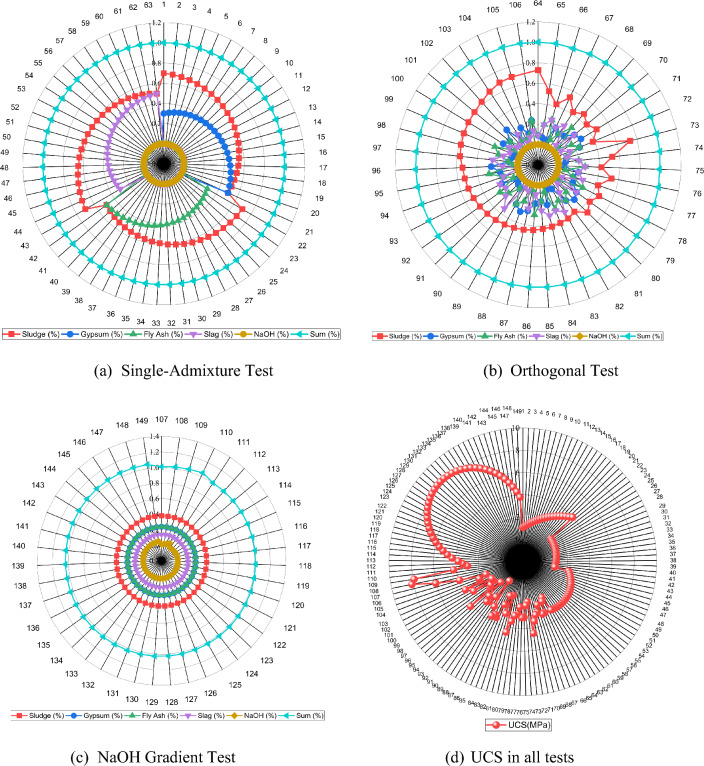
Sludge solidification tests: (**a**) single-admixture, (**b**) orthogonal, (**c**) NaOH gradient, and (**d**) UCS results.

The experimental dataset comprised 149 distinct material combinations, each systematically varying the proportions of sludge, fly ash, desulfurized gypsum, slag, and NaOH to investigate their influence on sludge solidification. The experimental program was designed^57^ to assess both individual and interactive effects of these components. Single-admixture tests were conducted to isolate the influence of each additive on the mechanical properties of the sludge, providing a baseline for understanding individual contributions. To examine the combined effects of multiple factors, orthogonal tests were performed, allowing efficient exploration of multi-factor interactions and identification of optimal mix ratios. Additionally, NaOH gradient tests were conducted to quantify the effect of varying alkali-activator concentrations on solidification performance. For all specimens, the UCS was measured at 28 days of curing using a Wance microcomputer-controlled pressure testing machine. Specimens were subjected to axial compression until failure without lateral support to determine their maximum compressive strength. The experimental results, including single-admixture, orthogonal, and NaOH gradient tests, are summarized in Fig. 3.

### Data preprocessing

**Fig. 4:**
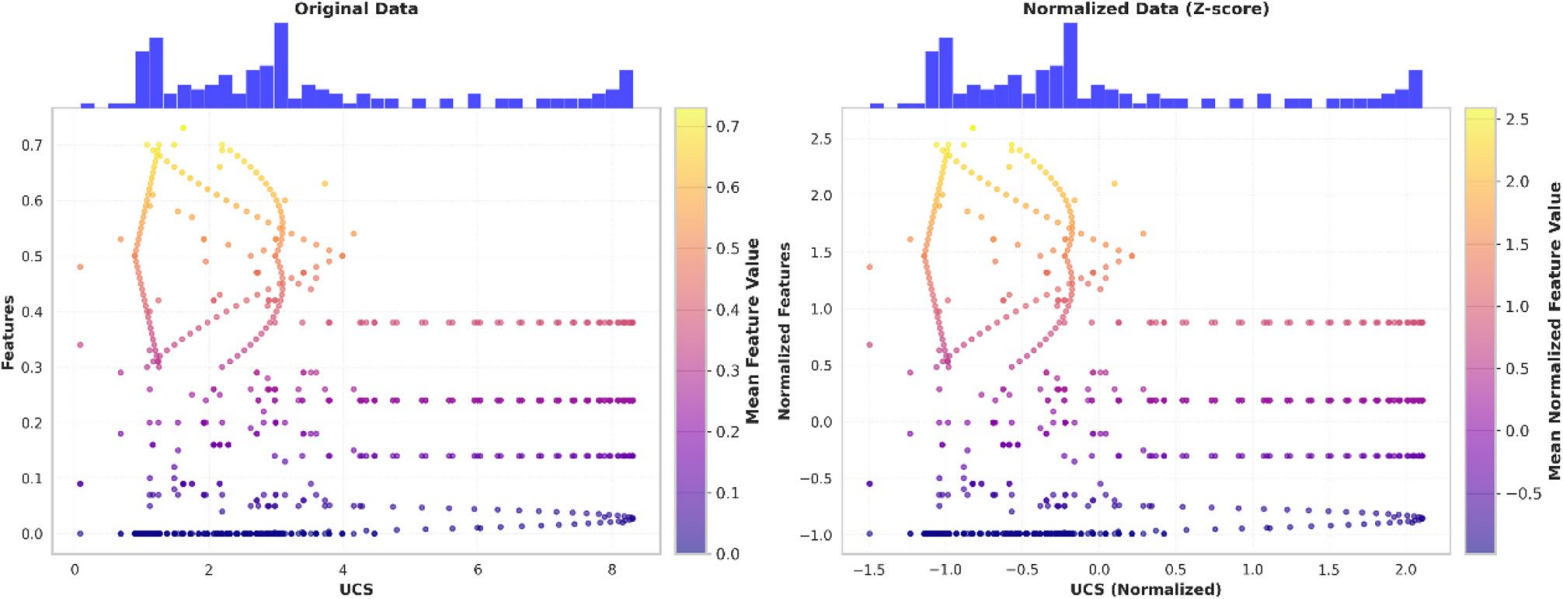
Original and normalized feature data with mean values using the Z-score method.

All experiments were conducted using Python 3.11. To eliminate scale-induced bias and ensure that all input variables contribute equally during predictive modeling and optimization, Z-score normalization was applied to the experimental dataset using the following equation:

1 $${X}^{\prime}=\frac{X-\mu }{\sigma }$$

where $\mu$ and $\sigma$ are the mean and standard deviation of the feature $X$. Features such as Sludge (%), Fly Ash (%), Gypsum (%), Slag (%), and NaOH (%). Post-normalization, all features became dimensionless with $\mu$ and $\sigma =1$. Figure 4 shows a comparison of the data distribution before and after this transformation. The preservation of scatter patterns confirms that normalization rescaled the feature magnitudes without altering their intrinsic correlations with the UCS response. This ensures that the optimization algorithm treats all chemical proportions with equal importance. Figure 4(a) displays the raw features against the UCS. The input variables (Sludge, Gypsum, Fly Ash, Slag, and NaOH) exist across vastly different numerical scales and units. Figure 4(b) illustrates the dataset after transformation into a dimensionless space with a mean (μ) of 0 and a standard deviation (σ) of 1. A visual comparison of the scatter patterns and the top-aligned histograms confirms that Z-score normalization rescales the magnitude of the variables without distorting the underlying correlations or the relative density of the data points.

**Fig. 5:**
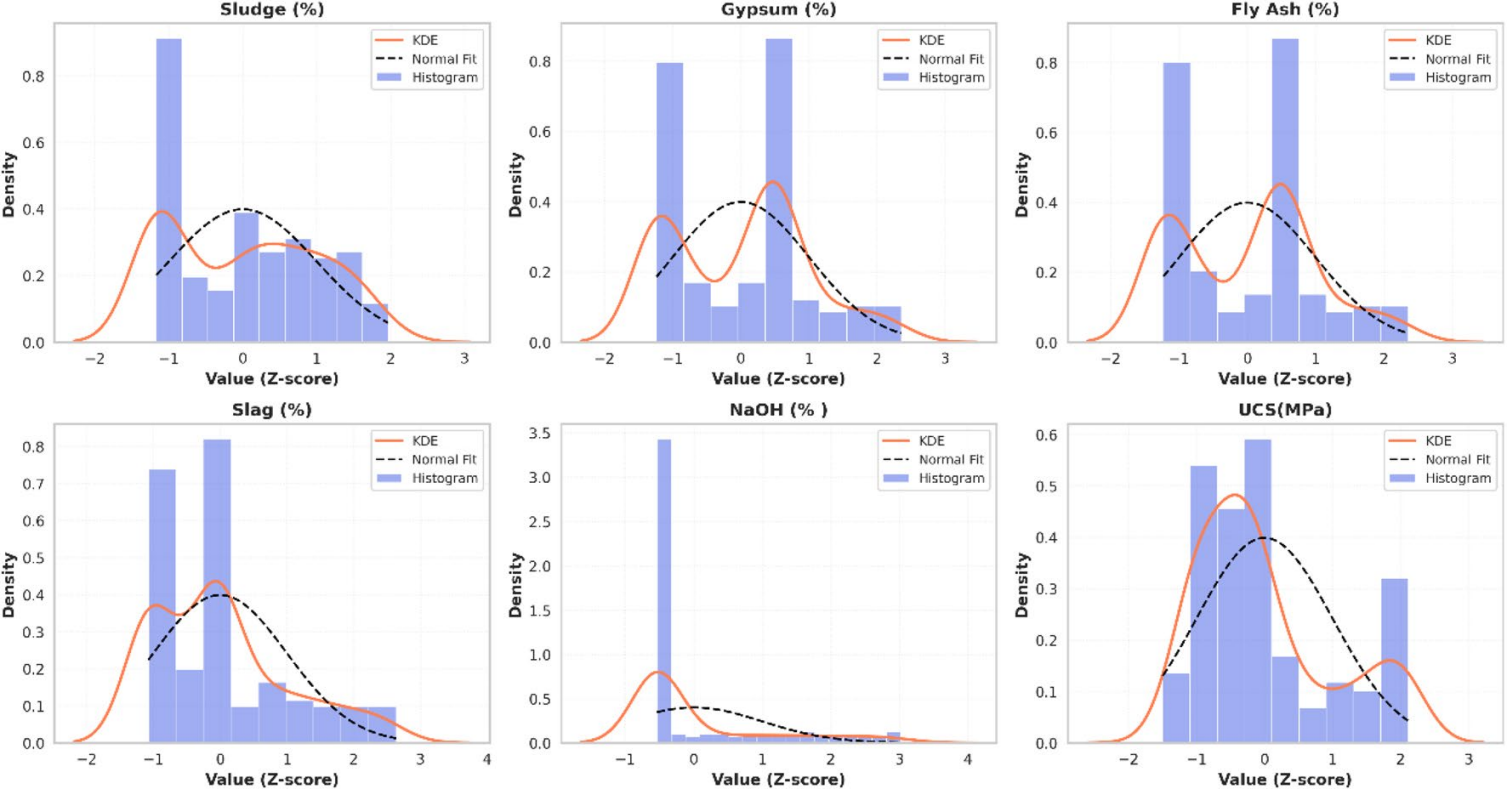
Distributional analysis of experimental features post-normalization.

Figure 5 illustrates the histograms and Kernel Density Estimation (KDE) distributions for all input parameters and the target UCS. The normalization process transformed the raw data into a dimensionless space with a mean (μ) of 0 and a standard deviation (σ) of 1. As shown in the Fig. 5, the KDE plots demonstrate the actual probability density of the experimental data. The close alignment between the KDE and the normal fit indicates that the dataset, following Z-score transformation, approximates a Gaussian distribution. This transformation eliminates numerical bias, stabilizes gradient descent during model training, and provides a robust foundation for subsequent maximum resistance optimization.

## Optimization model for modified solidified sludge mix design

The optimization model for the modified solidified sludge mix design aims to determine the ideal proportions of sludge, fly ash, gypsum, slag, and NaOH in order to maximize the Unconfined Compressive Strength (UCS), while adhering to the specified constraints. The objective is to maximize the UCS, which involves five decision variables: ${x}_{1},{x}_{2},{x}_{3},{x}_{4},{x}_{5}$. Here, ${x}_{1}$ is the sludge dosage (%), ${x}_{2}$ is the gypsum dosage (%), ${x}_{3}$ is the fly ash dosage (%), ${x}_{4}$ is the slag dosage (%), and ${x}_{5}$ is the NaOH concentration (%). The output of the optimization process is the UCS (MPa), denoted as $Y$, after 28 days of curing, which is the key performance indicator that the model aims to maximize. The optimization model can be formulated as follows:

2 $$\mathrm{Consider} \overrightarrow{x}:\overrightarrow{x}=[{x}_{1},{x}_{2},{x}_{3},{x}_{4},{x}_{5}]= [\mathrm{Sludge},\text{ Gypsum},\text{ Fly Ash},\text{ Slag},\text{ NaOH}]$$

3 $$\text{Maximize }f\left(\overrightarrow{x}\right): f\left(\overrightarrow{x}\right)=\mathrm{Y}=\text{UCS }\left({x}_{1}, {x}_{2}, {x}_{3}, {x}_{4}, {x}_{5}\right)$$

$$\mathrm{Constraints} g\left(\overrightarrow{x}\right):0.38\le {x}_{1}\le 0.73$$

$$0.0\le {x}_{2}\le 0.50$$

$$0.0\le {x}_{3}\le 0.50$$

$$0.0\le {x}_{4}\le 0.50$$

4 $$0.0\le {x}_{5}\le 0.051$$

## Hybrid WOA-ML models

**Fig. 6:**
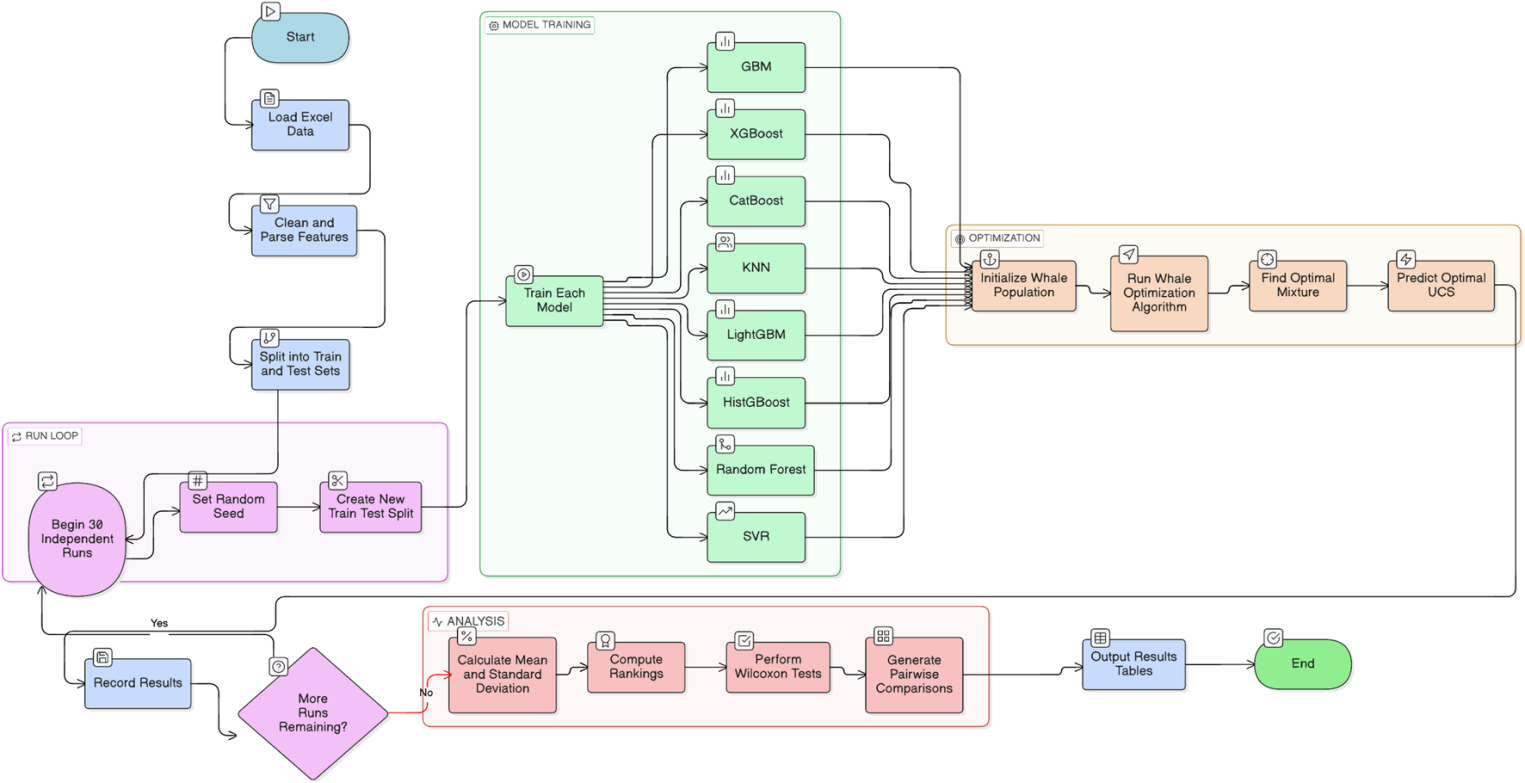
WOA-ML optimization workflow.

ML techniques have been increasingly applied in materials science to predict the mechanical behavior of waste-based cementitious systems, which are characterized by nonlinear interactions and multicollinearity among mixture components. These systems, particularly municipal sludge–based binders, exhibit complex chemical composition–property relationships that are difficult to capture using conventional empirical models. Figure 6 illustrates the system architecture for a 30-run independent simulation, integrating ensemble model training with the WOA–ML workflow for UCS prediction. To address these challenges and capture diverse learning paradigms suited to such complexity, eight ML regression algorithms, including Gradient Boosting Machine (GBM), Light Gradient Boosting Machine (LightGBM), Extreme Gradient Boosting (XGBoost), Categorical Boosting (CatBoost), k-Nearest Neighbors (KNN), Histogram-based Gradient Boosting (HistGBoost), Random Forest (RF), and Support Vector Regression (SVR), were employed to predict the UCS of slag, fly ash, desulfurization gypsum, and modified solidified sludge. This selection provides the methodological foundation for the subsequent mixture optimization.

### Gradient boosting machine (GBM)

GBM is an ensemble method that sequentially fits regression trees to minimize the residual errors of previous trees. The predicted UCS is given by the additive combination of $M$ trees:

5 $${\hat{Y}}_{\mathrm{UCS}}=\sum_{m=1}^{M} {\gamma }_{m}{h}_{m}(X)$$

where ${\hat{Y}}_{\mathrm{UCS}}\text{ is the}$ predicted UCS, ${h}_{m}\left(X\right)$ is the output of the $m$-th regression tree, ${\gamma }_{m}$ is the learning rate,$M$ is the number of trees. Each tree ${h}_{m}(X)$ models the residuals from the previous ensemble:

6 $${\text{r }}_{m}=y-\sum_{j=1}^{m-1} {\gamma }_{j}{h}_{j}(X)$$

GBM is particularly suitable for regression problems with nonlinear relationships and multidimensional dependencies. In the current study, hyperparameters ( $M,{\gamma }_{m}$, max depth) were optimized using the WOA.

### Extreme gradient boosting (XGBoost)

XGBoost extends GBM by incorporating regularization to prevent overfitting. Its prediction function is expressed as:

7 $${\hat{Y}}_{\mathrm{UCS}}=\sum_{m=1}^{M} {f}_{m}(X), {f}_{m}\in \mathcal{F}$$

where ${f}_{m}(X)$ is the $m$-th regression tree, and $\mathcal{F}$ represents the space of all possible trees. The objective function for XGBoost is:

8 $$\mathrm{Obj}=\sum_{i=1}^{N} l\left({y}_{i},{\hat{y}}_{i}\right)+\sum_{m=1}^{M}\Omega \left({f}_{m}\right),\Omega \left({f}_{m}\right)=\gamma T+\frac{1}{2}\lambda \sum_{j=1}^{T} {w}_{j}^{2}$$

where $l(\cdot )$ is the loss function, $T$ is the number of leaves, ${w}_{j}$ is the leaf weight, and $\gamma ,\lambda$ are regularization parameters. WOA optimizes the number of trees, learning rate, and maximum depth for UCS prediction.

### CatBoost

CatBoost is a gradient boosting algorithm optimized for categorical and numerical features. Its predictive formula is similar to GBM:

9 $${\hat{Y}}_{\mathrm{UCS}}=\sum_{m=1}^{M} {\gamma }_{m}{h}_{m}(X)$$

with the addition of ordered boosting to prevent target leakage when handling categorical variables. Each tree ${h}_{m}(X)$ is trained on permuted datasets, ensuring unbiased residual estimates. Hyperparameters such as iterations $(M)$, depth, and learning rate were optimized using WOA.

### Light gradient boosting machine (LightGBM)

LightGBM is a histogram-based gradient boosting model that grows trees leaf-wise, focusing on maximum.

reduction in loss per leaf. Its UCS prediction is given by:

10 $${\hat{Y}}_{\mathrm{UCS}}=\sum_{m=1}^{M} {\gamma }_{m}{h}_{m}\left({X}_{binned}\right)$$

where ${X}_{\mathrm{binned}}$ represents discretized input features. Leaf-wise growth allows LightGBM to efficiently handle large datasets while capturing complex nonlinear relationships. WOA was used to optimize the number of leaves, learning rate, and minimum child samples.

### k-Nearest neighbors (KNN)

KNN is a non-parametric regression algorithm that predicts UCS as the weighted average of the nearest $k$ neighbors:

11 $${\hat{Y}}_{\mathrm{UCS}}(x)=\frac{\sum_{i\in {\mathcal{N}}_{k}(x)} {w}_{i}{y}_{i}}{\sum_{i\in {\mathcal{N}}_{k}(x)} {w}_{i}}$$

where ${\mathcal{N}}_{k}(x)$ is the set of k nearest neighbors of sample $x$, ${w}_{i}$ is the weight of the $i$-th neighbor, ${y}_{i}$ is the true UCS of the $i$-th neighbor. The hyperparameters $k$, distance metric $p$, and weighting scheme were optimized using WOA to improve predictive accuracy.

### Histogram-based gradient boosting (HistGBoost)

HistGBoost is a gradient boosting algorithm that builds decision trees using histograms for faster training. The prediction for UCS is:

12 $${\hat{Y}}_{UCS}(x)=\sum_{t=1}^{T} {\alpha }_{t}{h}_{t}(x)$$

where $T$ is the number of trees, ${\alpha }_{t}$ is the tree weight, and ${h}_{t}(x)$ is the tree’s prediction. Hyperparameters were optimized using WOA to improve accuracy.

### Random forest (RF)

RF is an ensemble learning method that constructs multiple independent regression trees and predicts UCS as the average of their outputs:

13 $${\widehat{Y}}_{UCS}=\frac{1}{T}\sum_{t=1}^{T}{h}_{t}(X)$$

where $T$ is the total number of trees, ${h}_{t}(X)$ represents the prediction of the $t$-th tree, and $X$ is the input feature vector. Hyperparameters, including the number of trees, maximum depth, and minimum samples per leaf, were optimized using WOA to enhance UCS prediction accuracy.

### Support vector regression (SVR)

SVR is a kernel-based regression algorithm that estimates UCS by finding a function $f(X)$ that deviates from the true UCS values by at most $\epsilon$ while maintaining model flatness:

14 $$f(X)=\sum_{i=1}^{N}({\alpha }_{i}-{\alpha }_{i}^{*})K({X}_{i},X)+b$$

where $K$ is the kernel function, ${\alpha }_{i},{\alpha }_{i}^{*}$ are the Lagrange multipliers, $b$ is the bias term, and $N$ is the number of support vectors. In this study, the radial basis function (RBF) kernel was used, and hyperparameters including the penalty parameter $C$, kernel width $\gamma$ were optimized via WOA to maximize the predictive performance of UCS.

### Selection and justification of hybrid WOA-ML

The selected ML algorithms provide complementary strengths for predicting UCS of sludge-based mixtures. Ensemble-based methods (GBM, XGBoost, CatBoost, LightGBM, HistGBoost, RF) efficiently capture nonlinear and high-dimensional relationships, while instance-based (KNN) and kernel-based (SVR) models handle local patterns and smooth functional approximations. Coupling these models with WOA enables robust hyperparameter optimization across complex, nonconvex search spaces, enhancing predictive accuracy and supporting reliable mixture design.

## Optimization with different metaheuristic algorithm

Metaheuristic optimization is widely employed to efficiently explore complex solution spaces and approximate global optima in engineering applications. Unlike traditional methods, metaheuristics do not require derivative information and can handle non-linear, high-dimensional, and multi-objective problems. In this study, eight metaheuristic algorithms were implemented to optimize the mix design of sludge-based materials and predict their unconfined compressive strength (UCS). The algorithms include Traffic Jam Optimizer (TJO)^51^, Grey Wolf Optimizer (GWO)^50^, Whale Optimization Algorithm (WOA)^50^, Dream Optimization Algorithm (DOA)^52^, Particle Swarm Optimization (PSO)^47,48^, Genetic Algorithm (GA)^49^, Gazelle optimization algorithm (GOA)^53^, Young’s double-slit experiment optimizer (YDSE)^56^, Hiking Optimization Algorithm (HOA)^55^ and Octopus Optimization Algorithm (OOA)^54^. The selection of these algorithms is motivated by their distinct search strategies and capabilities in handling complex optimization problems. Each algorithm exhibits strengths in exploring large solution spaces, balancing exploration and exploitation, and avoiding premature convergence. Furthermore, these algorithms have been demonstrated to perform effectively in real-world engineering optimization problems, yielding near-optimal solutions for multi-component material systems.

### Selection and justification of metaheuristic optimization algorithms

**Fig. 7:**
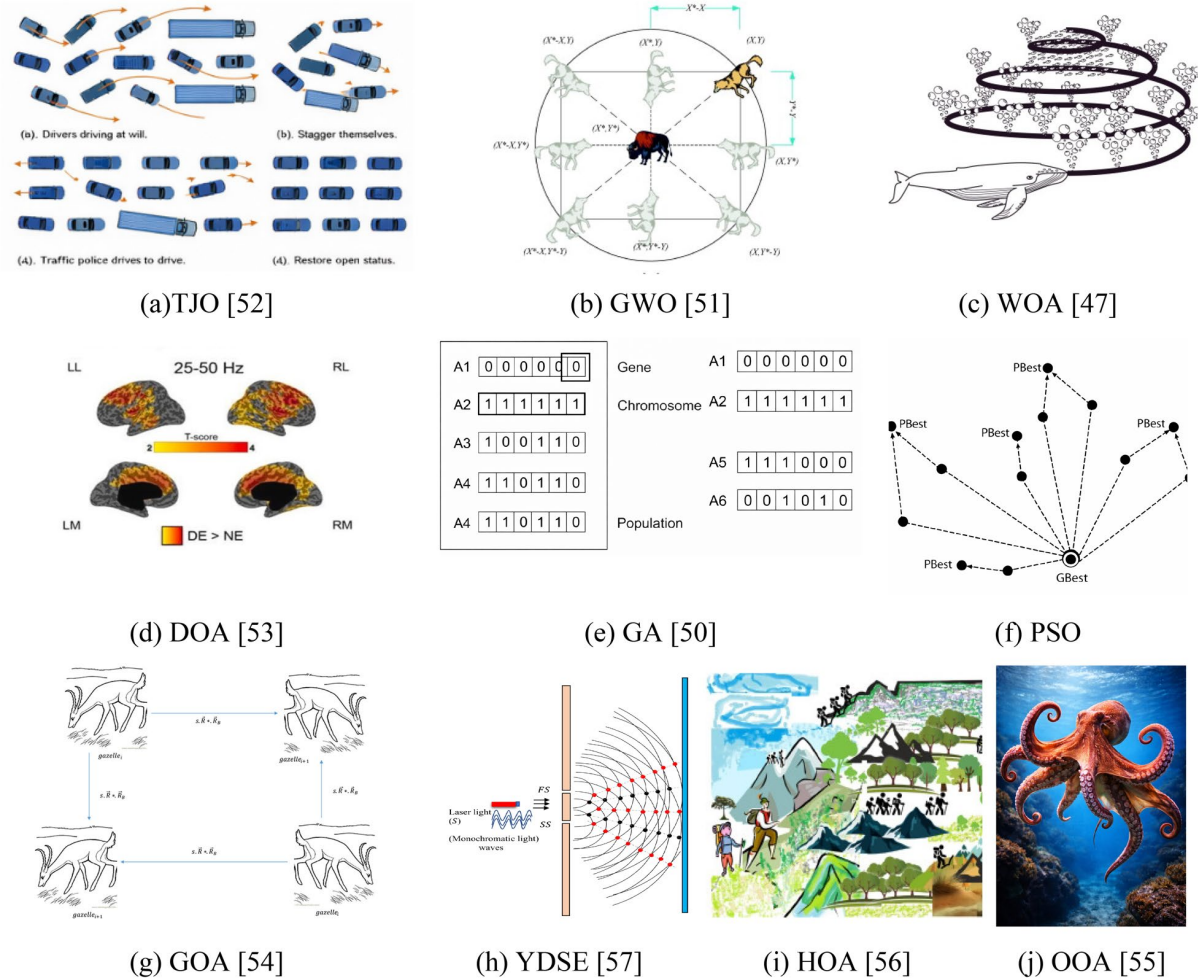
Diagram of the selected strategy process.

The selection of metaheuristic algorithms is crucial because each algorithm has different search mechanisms, strengths, limitations, and abilities to escape stagnation. An inappropriate choice may lead to local optima stagnation, excessive computational effort, or suboptimal prediction accuracy. Using different types of algorithms that employ various exploration–exploitation strategies can enhance robustness and reliability. Figure 7 shows the diagram of the selected strategy process. In this study, eight algorithms were chosen based on their complementary characteristics to maintain a balance between global search across the solution space and intensified local search in promising regions.

To address these challenges, eight metaheuristic algorithms were selected for this study: Traffic Jam Optimizer (TJO), Grey Wolf Optimizer (GWO), Whale Optimization Algorithm (WOA), Dream Optimization Algorithm (DOA), Particle Swarm Optimization (PSO), Genetic Algorithm (GA), Gazelle optimization algorithm (GOA), Young’s double-slit experiment optimizer (YDSE), Hiking Optimization Algorithm (HOA) and Octopus Optimization Algorithm (OOA). Each algorithm contributes unique strengths that complement the others, collectively enhancing robustness and solution reliability:

1. **Traffic jam optimizer (TJO)**: TJO utilizes a multi-phase search mechanism inspired by urban traffic flow transitions, enabling a smooth transition from global exploration to local refinement. This adaptive, phase-based search is particularly suited to the complex, nonlinear interactions among sludge, fly ash, gypsum, and NaOH.
2. **Grey wolf optimizer (GWO)**: GWO is suitable for optimizing sludge-based mixtures because it efficiently handles complex, nonlinear, and high-dimensional search spaces, such as those formed by varying proportions of sludge, fly ash, gypsum, slag, and NaOH. Its adaptive balance between exploration and exploitation prevents premature convergence to local optima, thereby increasing the likelihood of identifying the global optimum mixture.
3. **Whale optimization algorithm (WOA):** WOA mechanisms include shrinking encircling, spiral bubble-net feeding, and random prey search, which allow it to explore the global solution space broadly while exploiting promising regions near the current best solutions. This approach helps avoid local optima and increases the likelihood of finding the global optimum mixture. Additionally, WOA requires only a few tunable parameters, making it computationally efficient and practical for iterative optimization of mixture proportions, such as sludge, fly ash, gypsum, slag, and NaOH.
4. **Dream optimization algorithm (DOA):** DOA introduces stochastic perturbations inspired by human dream-like processes, maintaining population diversity and enhancing the ability to escape suboptimal regions. Its adaptive refinement strategies are beneficial for dynamic, non-linear material systems where solution landscapes are irregular.
5. **Particle swarm optimization (PSO):** PSO updates candidate solutions based on collective and individual experience, ensuring fast convergence toward high-fitness regions. Its simplicity and computational efficiency make it ideal for repeated runs necessary for statistical validation of optimized sludge compositions.
6. **Genetic algorithm (GA):** GA applies evolutionary operations (selection, crossover, mutation) to evolve solutions over successive generations. Its strength lies in diverse solution exploration, making it effective for combinatorial and discrete aspects of multi-component mix design.
7. **Gazelle optimization algorithm (GOA):** For optimizing material mixtures like sludge, fly ash, gypsum, and NaOH, GOA mimics two phases: exploitation, where gazelles graze or are stalked, and exploration, where they escape predators at high speeds (Lévy flight) to explore the solution space.
8. **Young’s double-slit experiment optimizer (YDSE):** YDSE optimizer treats bright and dark fringes as candidate solutions, with the central bright fringe as the near-optimal solution. By simulating interference, it balances global exploration in dark fringes and local exploitation in bright fringes, efficiently optimizing complex material mixtures like sludge, fly ash, gypsum, slag, and NaOH.
9. **Hiking optimization algorithm (HOA):** HOA models solutions as hikers navigating terrain, with slope affecting speed. Tobler’s Hiking Function sets velocity, and the sweep factor balances exploration and exploitation. Positions are updated iteratively, led by the fittest hiker, enabling efficient search of complex optimization problems like sludge–fly ash–gypsum–slag–NaOH mixtures.
10. **Octopus optimization algorithm (OOA):** OOA models solutions as octopuses navigating the search space, using hunting and mating behaviors to balance exploration and exploitation. Exploration simulates water-spraying escape and memory-guided motion, while exploitation mimics mating strategies for local intensification and diversity preservation. This approach is applied to optimize sludge, fly ash, gypsum, slag, and NaOH proportions, efficiently searching complex, nonlinear mixture landscapes.

The inclusion of these algorithms ensures comprehensive coverage of diverse search strategies, balancing global exploration and local exploitation, while maintaining computational feasibility. Moreover, employing multiple algorithms enables cross-validation of optimized mix proportions of modified solidified sludge, reducing the risk of algorithm-specific bias and enhancing the robustness of predicted UCS outcomes.

## Verification of the models

To validate each model, multiple metrics were used for assessment. In each model, ${y}_{i}$ denote the observed UCS for a sample as ${y}_{i}$, the predicted UCS as $,{\hat{y}}_{i}$, and the total number of samples as $N$. The coefficient of determination measures the proportion of variance in the observed UCS explained by the model:

15 $${R}^{2}=1-\frac{\sum_{i=1}^{N} {\left({y}_{i}-{\hat{y}}_{i}\right)}^{2}}{\sum_{i=1}^{N} {\left({y}_{i}-\overline{y}\right)}^{2}}$$

where $\overline{y}$ is the mean of the observed UCS values. The mean of the observed UCS values ranges from 0 to 1, with higher values indicating better predictive accuracy. Mean Absolute Error (MAE) measures the average absolute difference between predicted and observed UCS values.

16 $$\text{MAE }=\frac{1}{N}\sum_{i=1}^{N} \left|{y}_{i}-{\hat{y}}_{i}\right|$$

MAE provides an intuitive measure of the average magnitude of prediction errors, independent of their direction. To further assess error distribution, the Root Mean Squared Error (RMSE) quantifies the square root of the average squared differences:

17 $$RMSE =\sqrt{\frac{1}{N}\sum_{i=1}^{N} {\left({y}_{i}-{\hat{y}}_{i}\right)}^{2}}$$

This metric penalizes large errors more heavily than MAE, providing sensitivity to outliers. The Symmetric Mean Absolute Percentage Error (sMAPE) normalizes absolute prediction errors relative to the average of observed and predicted values:

18 $$s\text{MAPE }=\frac{100}{N}\sum_{i=1}^{N} \frac{2\left|{\hat{y}}_{i}-{y}_{i}\right|}{\left|{y}_{i}\right|+\left|{\hat{y}}_{i}\right|}$$

sMAPE provides a symmetric, percentage-based error measure that prevents bias toward over- or under-prediction. Systematic error is specifically captured by the Percent Bias (PBIAS), which measures the average tendency of predicted UCS values to be larger or smaller than observed values:

19 $$\text{PBIAS }=100\cdot \frac{\sum_{i=1}^{N} \left({\hat{y}}_{i}-{y}_{i}\right)}{\sum_{i=1}^{N} {y}_{i}}$$

The index of agreement assesses how well the predictions match observations, accounting for differences in both magnitude and variance:

20 $$\text{d }=1-\frac{\sum_{i=1}^{N} {\left({\hat{y}}_{i}-{y}_{i}\right)}^{2}}{\sum_{i=1}^{N} {\left(\left|{\hat{y}}_{i}-\overline{y}\right|+\left|{y}_{i}-\overline{y}\right|\right)}^{2}}$$

The index ranges from 0 (no agreement) to 1 (perfect agreement) and is sensitive to both systematic and random errors.

## Discussion and results

### Sensitivity analysis of the WOA‑ML parameters

A sensitivity analysis was conducted to investigate the influence of control parameters on the performance of the proposed WOA–ML framework. Parameter ranges were selected based on the literature^46^. The objectives of this sensitivity analysis are threefold: (i) to determine suitable operating ranges for each parameter, (ii) to identify parameters that can be fixed at default values without significantly affecting performance, and (iii) to justify the parameter settings adopted in the main experiments. The analysis considered population size $N$, maximum iterations $T$, inertia weights ${w}_{i}$ and ${w}_{j}$, mutation factor ${\gamma }_{m}$, convergence parameter $\gamma$, regularization coefficient $\lambda$, and adaptive learning rate ${\alpha }_{t}$.

Table 2 presents the results for population sizes ranging from 10 to 50, while keeping the maximum iterations fixed at 200. The average UCS increases from 8.2354 MPa (N = 10) to a peak of 8.2799 MPa at *N* = 30, then slightly declines. The standard deviation remains low (≤ 0.0057) across all settings, indicating consistent performance. The number of evaluations required to reach the best solution rises almost linearly with population size, from 1990 at *N* = 10 to 9650 at N = 50. The highest average UCS and moderate computational cost suggest *N* = 30 as a balanced choice for subsequent analyses.

**Table 2:** Sensitivity analysis on population size.

Parameter *N*	10	20	**30**	40	50
Best UCS (MPa)	8.2411	8.2483	8.2853	8.2372	8.2297
Worst UCS (MPa)	8.2297	8.2384	8.2744	8.2272	8.2268
Avg UCS (MPa)	8.2354	8.2334	8.2799	8.2322	8.2283
Std UCS (MPa)	0.0057	0.0050	0.0054	0.0050	0.0015
Evals for Best	1990	3460	5790	7440	9650
Avg Evals	1920	3670	5160	7080	8600

Aa shown in Table 3, varying the maximum iterations from 50 to 400, shows that the average UCS improves substantially from 8.1629 MPa at *T* = 50 to 8.2345 MPa at *T* = 200, and further to 8.2385 MPa at *T* = 300. However, the standard deviation spikes to 0.0352 at *T* = 300, indicating greater variability. The run with *T* = 200 exhibits an exceptionally low standard deviation (0.0001) while still achieving a high average UCS (8.2345 MPa). The evaluations for best increase with *T*, as expected. Considering both solution quality and stability, *T* = 200 appears sufficient; increasing beyond 200 yields marginal gains with increased variability.

**Table 3:** Sensitivity analysis on maximum number of iterations.

Parameter *T*	50	100	**200**	300	400
Best UCS (MPa)	8.2012	8.2331	8.2346	8.2337	8.1914
Worst UCS (MPa)	8.1245	8.2165	8.2344	8.2133	8.1777
Avg UCS (MPa)	8.1629	8.2248	8.2345	8.2385	8.1846
Std UCS (MPa)	0.0384	0.0083	0.0001	0.0352	0.0068
Evals for Best	1440	2700	5370	8820	11,940
Avg Evals	1470	2835	5535	8700	11,880

The effect of ${w}_{i}$ is shown in Table 4. When ${w}_{i}$ is 10, the performance is poor (best UCS 6.2050 MPa). For ${w}_{i}$ ≥50, the best UCS ranges between 8.1833 and 8.3217 MPa, with the maximum at ${w}_{i}$=150 (8.3217 MPa). The average UCS fluctuates around 8.10 MPa, and standard deviations are relatively high (~ 0.2. The evaluation counts are similar across settings. A value of ${w}_{i}$=150 can be recommended as it yields the highest observed UCS.

**Table 4:** Sensitivity analysis results for ${w}_{i}$.

Parameter ${w}_{i}$	10	50	100	150	200	300	400	500
Best UCS (MPa)	6.2050	8.1833	8.2898	8.3217	8.2990	8.3054	8.2951	8.3008
Worst UCS (MPa)	6.1121	7.7844	7.5384	7.7562	7.8185	7.5999	7.8069	7.7234
Average UCS (MPa)	6.1957	7.9916	8.1152	8.0482	8.1224	8.0912	8.1002	8.1288
Standard Deviation	0.0279	0.1597	0.2179	0.1941	0.1892	0.2306	0.1777	0.1909
Evals for Best	5820	5670	5760	5970	6000	4950	5670	5850
Average Evaluations	5565	5403	5430	5322	5745	5343	5490	5091

Parameter ${w}_{j}$ was tested from 1 to 50, as shown in Table 5. A value of 1 is clearly insufficient, yielding a best UCS of 7.5949 MPa. Increasing ${w}_{j}$ to 5 improves the best UCS to 8.2792 MPa, while ${w}_{j}$ ≥ 10 produces nearly identical best UCS values (≈ 8.3049 MPa). The standard deviation decreases sharply after ${w}_{j}$ = 10, from 0.1798 at ${w}_{j}$ = 5 to 0.0400 at ${w}_{j}$ = 10, indicating enhanced model stability. Values of ${w}_{j}$ above 10 provide no further improvement; therefore, ${w}_{j}$ = 10 is considered optimal.

**Table 5:** Sensitivity analysis results for ${w}_{j}$.

Parameter ${w}_{j}$	1	5	10	20	30	40	50
Best UCS (MPa)	7.5949	8.2792	8.3049	8.3049	8.3049	8.3049	8.3049
Worst UCS (MPa)	5.6580	7.7199	8.1812	8.0978	8.1307	8.0792	7.9808
Average UCS (MPa)	7.1125	8.1042	8.2304	8.2527	8.2439	8.2247	8.2101
Standard Deviation	0.5437	0.1798	0.0400	0.0572	0.0516	0.0634	0.0922
Evals for Best	5190	6000	5100	5790	6000	4740	5790
Average Evaluations	5169	5730	5652	5112	5523	5532	5895

The γ parameter, varied from 0.0 to 1.5, as shows in Table 6. The best UCS values fluctuate between 8.2541 and 8.3018 MPa, while the average values range from 8.0464 to 8.1032 MPa. The standard deviations remain similar (approximately 0.17–0.22). Therefore, this parameter has a negligible impact on the optimization outcome, and the default value of 1.0 is acceptable.

**Table 6:** Sensitivity analysis results for $\gamma$.

Parameter $\gamma$	0.0	0.2	0.5	0.8	1.0	1.5
Best UCS (MPa)	8.2838	8.2735	8.2926	8.2541	8.2813	8.3018
Worst UCS (MPa)	7.8253	7.8233	7.6128	7.8065	7.7471	7.8603
Average UCS (MPa)	8.1017	8.1032	8.0959	8.0464	8.0845	8.0529
Standard Deviation	0.1729	0.1888	0.2167	0.1840	0.1923	0.1660
Evals for Best	5970	4470	5670	5850	5940	5820
Average Evaluations	5214	5145	5232	5619	5052	5622

As shown in Table 7, increasing ${\gamma }_{m}$ from 0.5 to 2.5 results in a gradual decline in performance. The best UCS decreases from 7.8190 to 7.4233 MPa, and the average UCS drops from 7.7562 to 7.3677 MPa. Meanwhile, the standard deviation increases, indicating reduced stability at higher ${\gamma }_{m}$ values. These results suggest that lower ${\gamma }_{m}$ values, particularly around 0.5, yield better and more stable performance.

**Table 7:** Sensitivity analysis results for ${\gamma }_{m}$.

Parameter ${\gamma }_{m}$	0.5	1.0	1.5	2.0	2.5
Best UCS (MPa)	7.8190	7.6661	7.5560	7.4691	7.4233
Worst UCS (MPa)	7.5967	7.4369	7.2221	7.0978	6.9385
Average UCS (MPa)	7.7562	7.5946	7.4178	7.3716	7.3677
Standard Deviation	0.0817	0.0818	0.1209	0.1398	0.1446
Evals for Best	6000	5610	5880	5640	6030
Average Evaluations	5274	5607	5316	5703	5526

The λ parameter was tested from 1 to 5, as shown in Table 8. The best UCS declines slightly from 8.2752 MPa at λ = 1 to 8.2265 MPa at λ = 5, while the average UCS increases from 8.0131 to 8.1831 MPa. The standard deviation drops markedly at λ = 3 and λ = 5, indicating more stable results. The improvement in average UCS and reduced variability suggest that using a moderate number of features (e.g., 3) may be preferable, even if the absolute best value is slightly lower.

**Table 8:** Sensitivity analysis results for $\lambda$.

Parameter $\lambda$	1	2	3	4	5
Best UCS (MPa)	8.2752	8.2588	8.2444	8.2343	8.2265
Worst UCS (MPa)	7.7717	7.7589	8.1216	7.9286	8.0979
Average UCS (MPa)	8.0131	8.0542	8.1702	8.0986	8.1831
Standard Deviation	0.1780	0.1833	0.0392	0.1134	0.0444
Evals for Best	5700	5370	5460	5970	5610
Average Evaluations	4785	5220	5451	5478	5316

The ${\alpha }_{t}$ parameter was varied from 0.05 to 0.50, as shown in Table 9. The best UCS decreases gradually from 8.2303 MPa at ${\alpha }_{t}$ = 0.05 to 8.1177 MPa at ${\alpha }_{t}$ = 0.50, while the average UCS peaks at ${\alpha }_{t}$= 0.30 (8.1451 MPa) and then declines. The standard deviation is lowest at ${\alpha }_{t}$ = 0.30 (0.0324). These results indicate that using a smaller fraction of samples per tree tends to yield higher peak UCS, whereas a fraction of around 0.30 provides the most reliable performance on average.

**Table 9:** Sensitivity analysis results for ${\alpha }_{t}$.

Parameter ${\alpha }_{t}$	0.05	0.10	0.20	0.30	0.40	0.50
Best UCS (MPa)	8.2303	8.2466	8.2112	8.1821	8.1513	8.1177
Worst UCS (MPa)	7.6952	7.9823	7.8242	8.0742	7.9416	7.9967
Average UCS (MPa)	8.0281	8.1181	8.1394	8.1451	8.0809	8.0770
Standard Deviation	0.1865	0.0895	0.1087	0.0324	0.0746	0.0400
Evals for Best	3990	5280	5820	4470	5850	5400
Average Evaluations	4377	5427	5733	5238	5244	5739

### Hybrid WOA-ML performance

The performance of the optimized models is summarized in Table 10. All WOA-ML approaches used to forecast this variant of sludge performed well and are therefore competitive with each other, as indicated in Table 10. While the primary evaluation employed repeated tenfold cross-validation, a 75/25 train-test split was found to provide the best balance in terms of model performance. Among the models analyzed, GBM demonstrated the best predictive capability, achieving an R^2^ of 0.987 and the lowest prediction errors, with an RMSE of 0.256 MPa, an MAE of 0.158 MPa, a low sMAPE of 6.30%, and minimal bias. The Index of Agreement (d = 0.997) confirms the excellent correlation between predicted and measured UCS results. CatBoost and XGBoost also achieved high accuracy, both exceeding 0.97 for R^2^ and above 0.99 for the Index of Agreement, demonstrating their robustness when modeling nonlinear relationships between binder composition and UCS. LightGBM and HistGBoost, on the other hand, achieved significantly lower levels of accuracy, with R^2^ below 0.90, accompanied by higher error levels, RMSE ≈ 0.72 MPa, MAE ≈ 0.51 MPa in relation to the other tested models. The relatively high levels of sMAPE and PBIAS for LightGBM and HistGBoost highlighted an opportunity for improvement around UCS prediction in under- or over-estimate areas for some test samples. W-RF and W-SVR also demonstrated strong predictive performance in UCS prediction. Among the WOA-ML approaches, the KNN method showed moderate levels of performance. Overall, these results indicated that, compared to other WOA-ML methods, tree-based ensemble methods such as GBM, CatBoost, and XGBoost provide for much more accurate UCS predictions for sludge-based composition materials with almost no bias.

**Table 10:** Performance metrics for eight WOA-ML models predicting UCS (MPa).

Model	R^2^	RMSE	MAE	sMAPE (%)	PBIAS (%)	Index of Agreement (d)
GBM	0.987	0.256	0.158	6.30	1.65	0.997
LightGBM	0.896	0.723	0.511	17.12	-4.24	0.969
XGBoost	0.971	0.382	0.191	7.80	-0.23	0.993
CatBoost	0.982	0.301	0.175	7.09	0.48	0.995
KNN	0.961	0.443	0.243	10.08	-0.26	0.990
HistGBoost	0.897	0.722	0.513	16.36	-3.34	0.969
RF	0.9726	0.1807	0.3716	7.0525	0.6764	0.9931
SVR	0.9609	0.2583	0.4439	10.6137	1.0963	0.9900

**Fig. 8:**
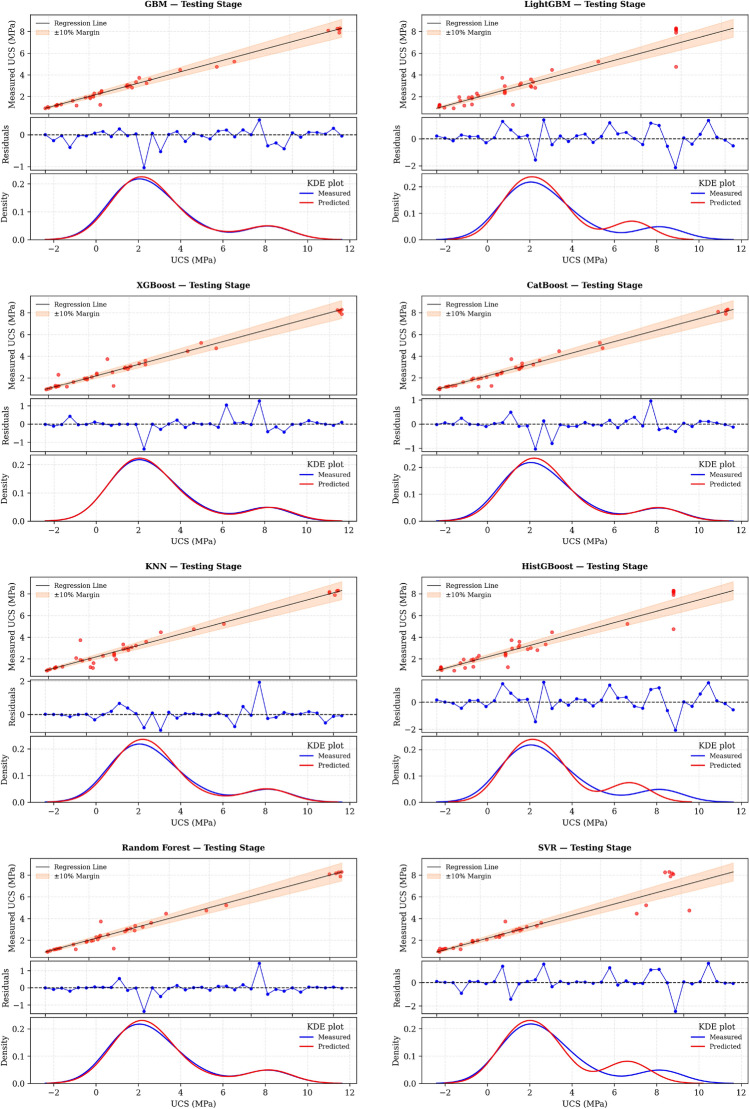
Testing performance of WOA-ML models: measured–predicted UCS, residuals, and KDE distributions.

Figure 8 shows the performance assessment of ML models during testing, showing regression plots comparing measured and predicted UCS values, residual distributions, and KDE probability profiles for all applied algorithms. The results in Fig. 8 indicate that the optimized boosting models (XGBoost, CatBoost, and GBM) closely match the experimental data, with the majority of points lying within a ± 10% error range. The associated Kernel Density Estimation (KDE) plots further confirm that the predicted strength distributions closely resemble the observed UCS distributions, demonstrating that the models have effectively captured the chemical interactions among slag, fly ash, and gypsum stabilizers.

**Fig. 9:**
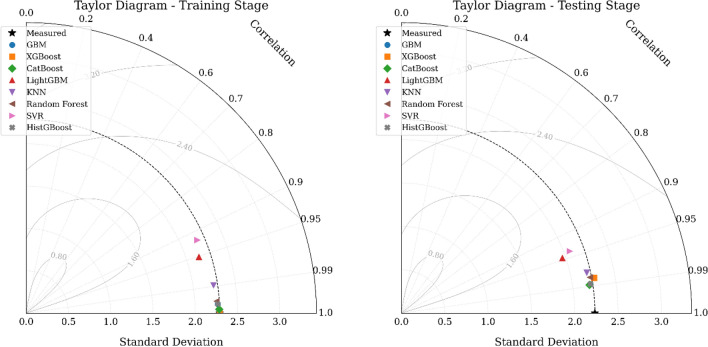
Taylor diagram of WOA-ML model performance in training and testing phases.

To evaluate and compare the performance of the WOA-ML models in predicting UCS, a Taylor diagram was employed, which simultaneously visualizes correlation, standard deviation, and centered RMSE (cRMSE) for a comprehensive assessment of model accuracy and consistency. The results for both training and testing stages are shown in Fig. 9. As illustrated in Fig. 9, the XGBoost model achieved the best performance in the training stage, positioned closest to the reference point of measured UCS, with a low cRMSE and high correlation, indicating strong agreement with experimental values. LightGBM and CatBoost also performed well, with slightly higher cRMSE values, while KNN, SVR, Random Forest, and HistGBoost showed larger deviations. During the testing stage, XGBoost again demonstrated the highest accuracy and stability, maintaining a low cRMSE and high correlation with measured data. LightGBM and CatBoost remained competitive, whereas KNN, SVR, Random Forest, and HistGBoost exhibited increased prediction errors, indicating lower generalization capacity. Overall, the Taylor diagram confirms that XGBoost consistently outperforms other WOA-ML models across both training and testing phases.

**Fig. 10:**
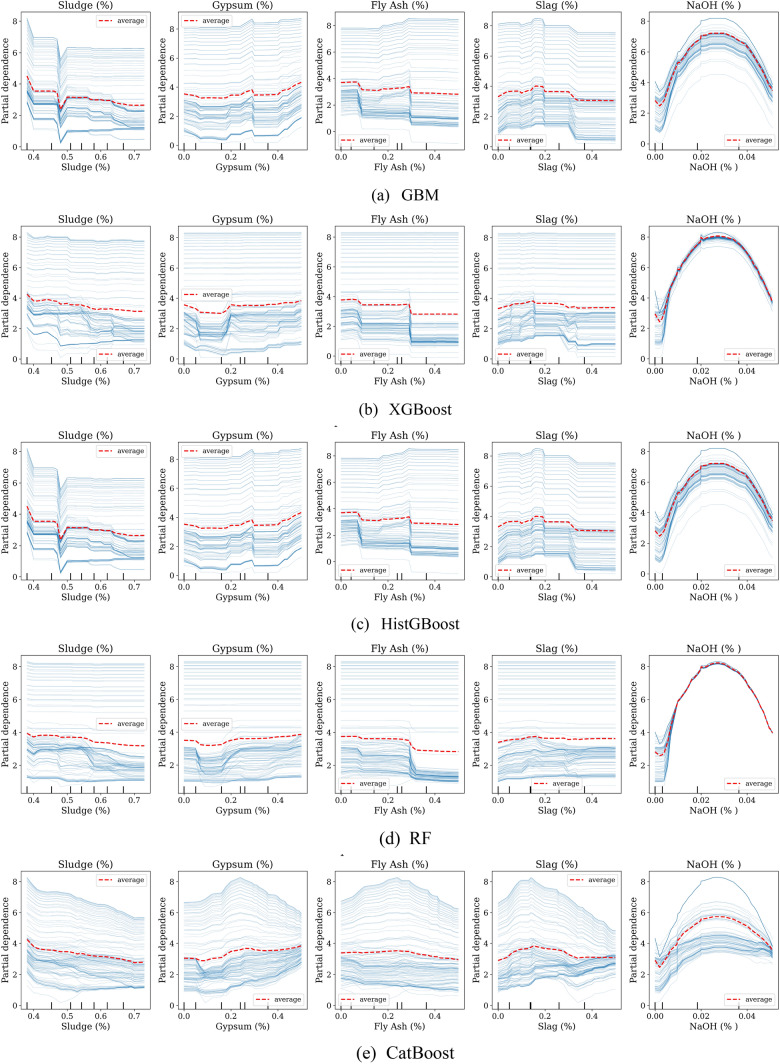
PDP–ICE plots of input variables on UCS of modified sludge.

Figure 10 illustrates the partial dependence and individual conditional expectation (PDP–ICE) plots for the five input variables (sludge, gypsum, fly ash, slag, and NaOH) derived from multiple machine learning models. Based on the PDP results shown in Fig. 10, each feature exhibits a distinct influence on the UCS of modified sludge. The main observations are as follows:

- **Effect of sludge content:** Sludge generally has a negative effect on UCS, with higher proportions (0.4–0.7) reducing performance, especially in GBM, HistGBM, and RF models.
- **Effect of gypsum content:** Gypsum moderately enhances UCS up to 0.25–0.35, after which gains plateau. XGBoost and CatBoost show smoother trends, while tree ensembles exhibit minor stepwise variations.
- **Effect of fly ash content:** Fly ash shows a threshold effect; below 0.20–0.25, UCS is stable, but higher contents reduce performance, notably in RF and XGBoost, suggesting diminished binding efficiency.
- **Effect of slag content:** Slag positively influences UCS at low to moderate levels (0.10–0.27) with stabilization or slight decrease at higher dosages.
- **Effect of NaOH concentration:** NaOH exhibits a nonlinear unimodal effect, increasing UCS up to 0.02–0.03 and declining at higher concentrations, indicating an optimal activator dosage.

### Uncertainty quantification in masing learning

**Fig. 11:**
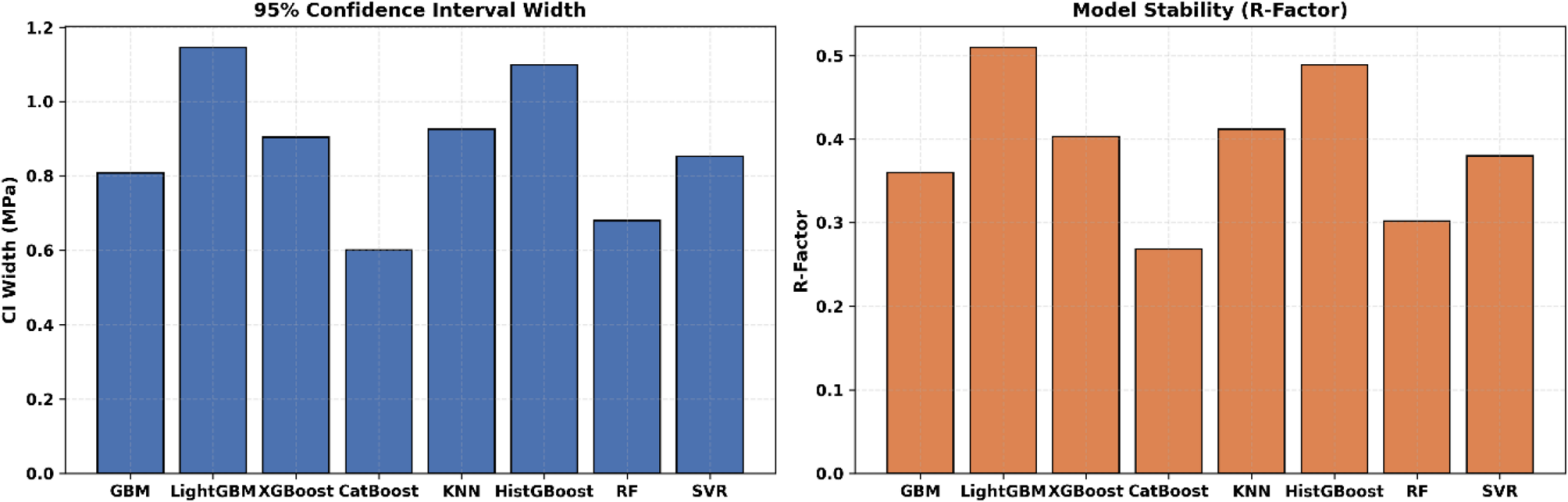
Uncertainty quantification metrics (CI₉₅% and R-factor) for eight ML models.

To further evaluate the reliability of the developed ML models, prediction uncertainty was quantified using the 95% confidence interval (CI₉₅%), CI width, and the R-factor. The CI₉₅% represents the range within which 95% of the predicted UCS values are expected to fall, while the CI width reflects the dispersion of prediction uncertainty. The R-factor, defined as the ratio of the average CI width to the standard deviation of the observed data, provides a normalized measure of uncertainty, which lower values indicating more reliable and stable predictions. Figure 11 shows that all models produced relatively narrow confidence intervals, indicating acceptable levels of uncertainty. Among them, HistGBoost exhibited the narrowest CI₉₅% with a CI width of 1.170 and the lowest R-factor (0.521), indicating the most compact uncertainty band. However, this reduced uncertainty must be interpreted alongside its comparatively lower predictive accuracy reported earlier. In contrast, GBM, XGBoost, and KNN showed slightly wider CI widths (1.450–1.472) with R-factors around 0.65, reflecting a moderate increase in uncertainty but accompanied by substantially higher predictive accuracy. CatBoost achieved a balanced performance, combining a relatively narrow CI width (1.411) with a low R-factor (0.628), which aligns well with its strong accuracy metrics. LightGBM demonstrated moderate uncertainty, with a CI width of 1.287 and an R-factor of 0.573. Overall, the uncertainty analysis confirms that GBM, CatBoost, and XGBoost provide an optimal trade-off between prediction accuracy and uncertainty, whereas HistGBoost, despite exhibiting the lowest uncertainty, may sacrifice predictive performance.

**Fig. 12:**
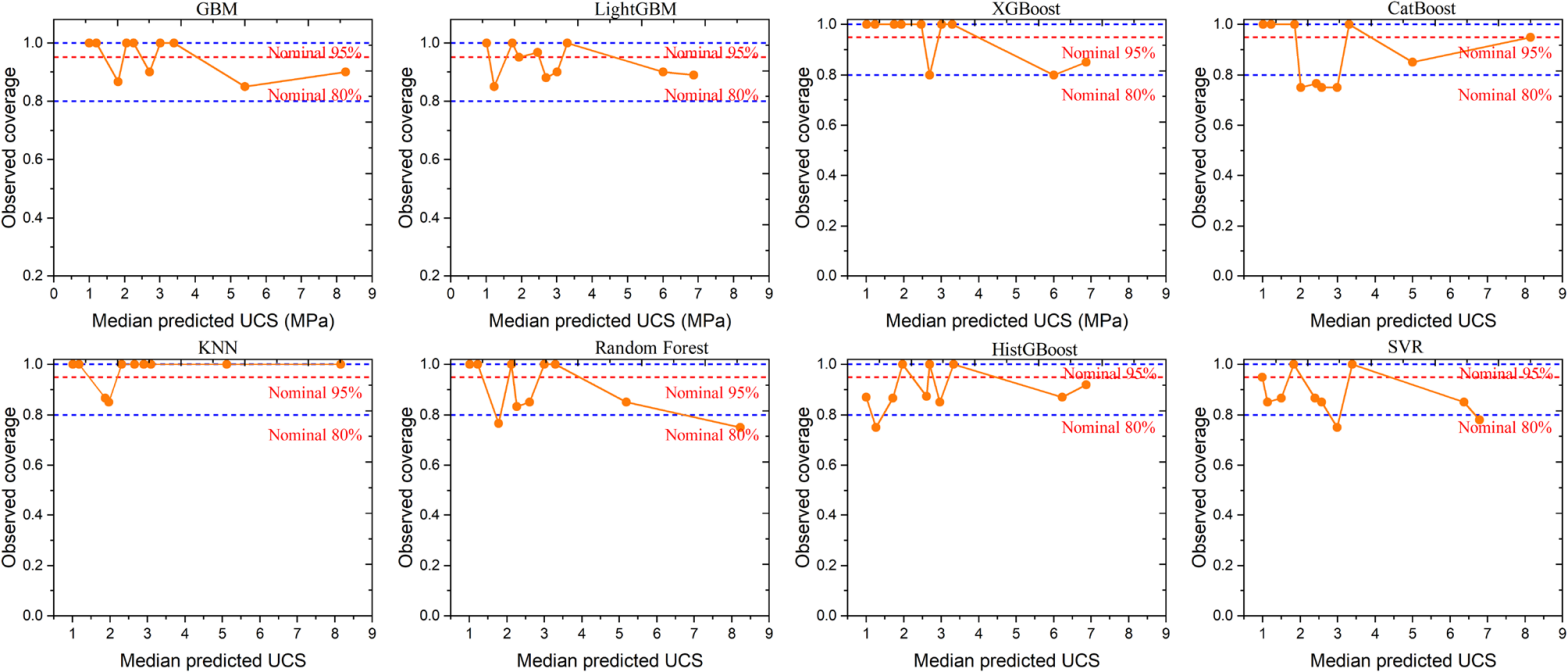
WOA-ML calibration curves with 95% CIs and nominal lines.

Figure 12 shows 95% prediction intervals using bootstrap resampling to evaluate model reliability. Empirical coverage, the proportion of true UCS values within intervals, was calculated, ideally near 0.95. Coverages ranged from 0.84 to 0.95. XGBoost showed the best calibration (coverage = 0.950), while SVR under covered (0.840). Figure 12 presents calibration curves with observed coverage in 10 bins of median predicted UCS, including 95% Wilson score CIs. Observed coverage value was align with the 0.95 nominal line for perfect calibration. XGBoost’s curve stays within confidence bands, indicating reliable uncertainty across ranges. SVR shows under-coverage at low UCS an d over-coverage at high, implying inconsistent uncertainty. Thus, XGBoost excels in accurate predictions (R^2^, MAE) and calibrated uncertainties.

### SHAP sensitivity analysis

**Fig. 13:**
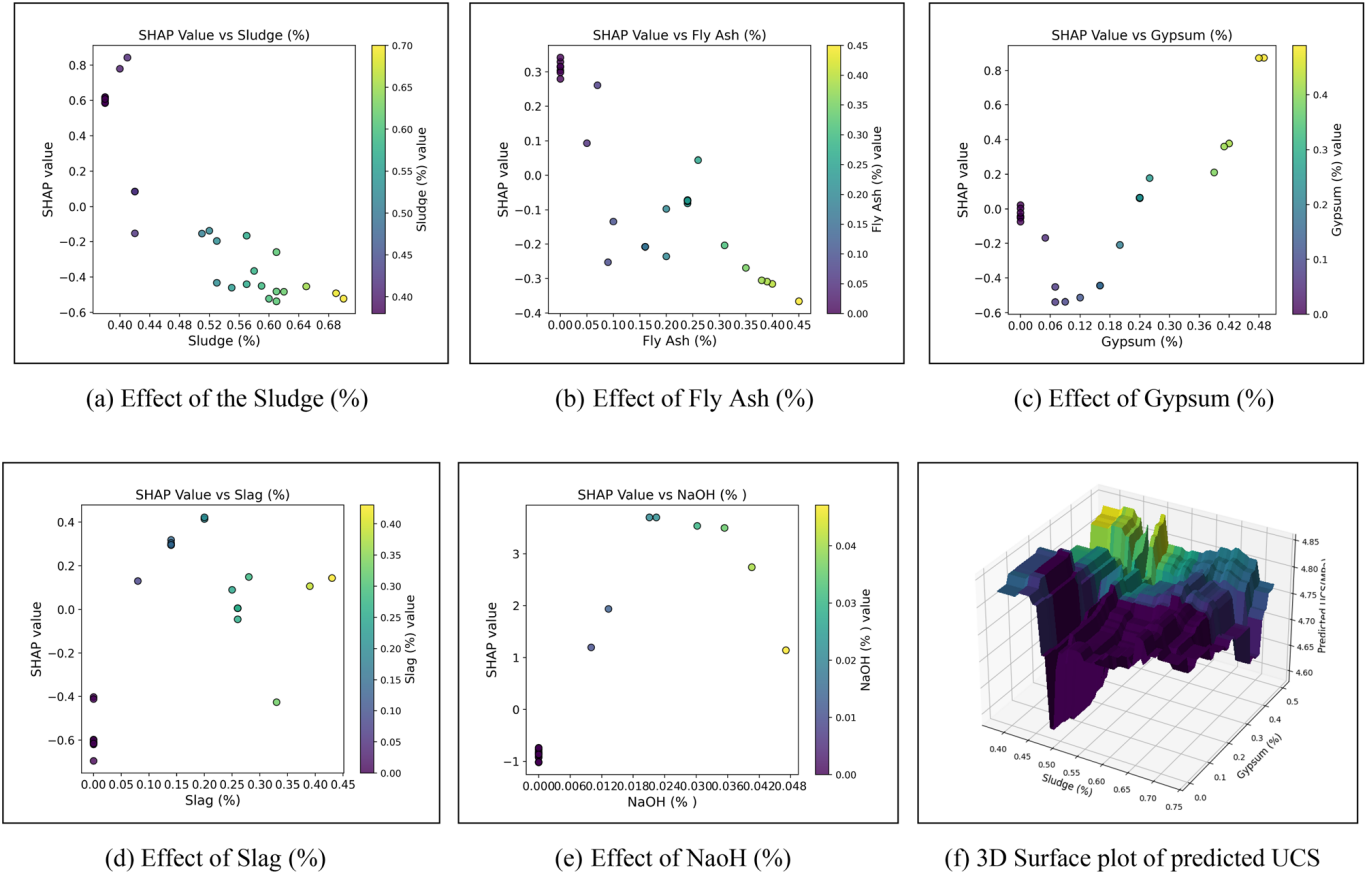
SHAP analysis of input components on predicted UCS.

To interpret the model’s behavior, SHAP analysis was used to analyze the local and global influence of each feature on the predicted UCS. Figure 13 shows the sensitivity analysis of each feature’s impact on UCS. Figure 13(a) shows a significant negative correlation with SHAP values and sludge concentration above 45%, while lower sludge content (approximately 38%) corresponds to positive SHAP values that contribute to higher UCS. Concentrations exceeding 50% lead to a sharp decline in strength contribution. Figure 13(c) demonstrates a strong positive correlation between gypsum content and SHAP value. As gypsum content increases from 0% to approximately 50%, the SHAP value rises from negative to about 0.8. The alkali-activator, NaOH (%) in Fig. 13(e), demonstrates a threshold effect where very low concentrations (near 0%) have a strong negative impact on strength. As the NaOH percentage increases toward 2%, the SHAP value increases significantly before plateauing, indicating that further increases beyond this point do not substantially enhance strength, thus marking an optimal range for the sludge-based material process. Both Fly Ash in Fig. 13(b) and Slag in Fig. 13(d) show fluctuating but generally positive contributions within specific ranges, with fly ash providing peak positive contributions at lower percentages (around 5%) and slag showing optimal performance between 15 and 25%. Figure 13(f) shows the 3D surface plot colored by SHAP values, illustrating the interaction between Sludge and Gypsum and their influence on the predicted UCS. The height of the surface represents the predicted UCS values across varying concentrations of Sludge and Gypsum, while the color intensity indicates the magnitude of the combined SHAP values for these two features. Areas with higher or lower SHAP values highlight areas where the interaction between Sludge and Gypsum has a more significant positive or negative impact on the UCS prediction relative to the model’s baseline. This visualization enables the identification of specific combinations of these two features that most strongly influence the UCS.

**Fig. 14:**
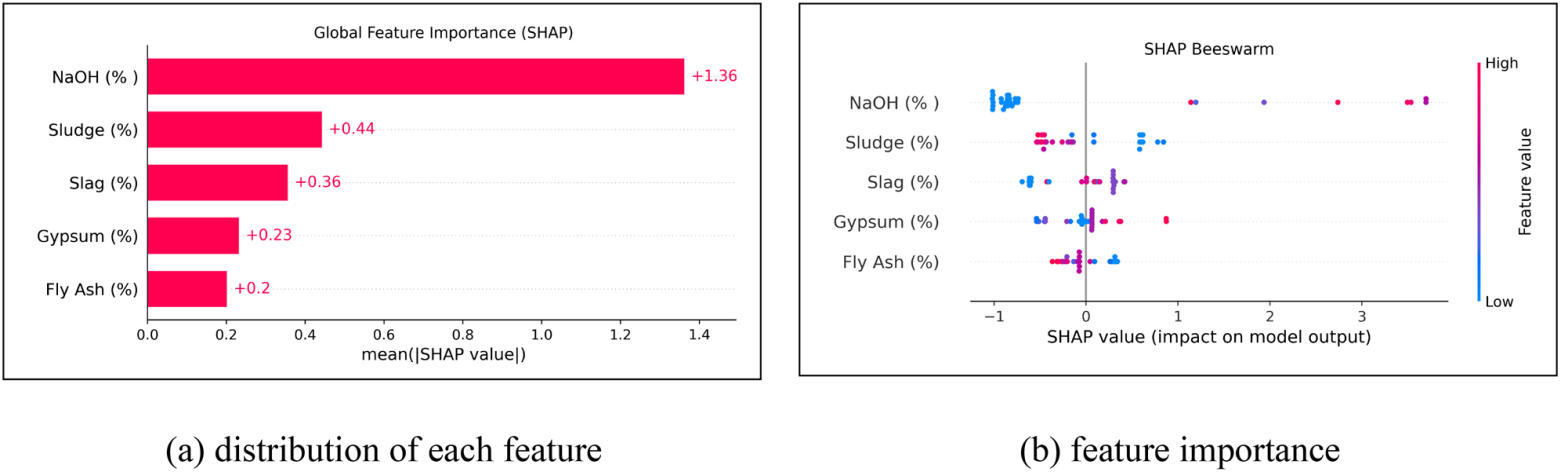
Global impact and feature importance of geometric parameters based on SHAP values.

Figure 14 presents the global impact and the distribution of effects across all features. To assess the relative importance of these features in maximizing UCS, a SHAP global analysis was conducted, as shown in Fig. 14(a), where each bar represents the mean absolute SHAP value for a given feature. The results indicate that NaOH has the most significant impact on the model’s predictions. Other influential features include Sludge, Slag, and Gypsum, reflecting their substantial contributions to chemical reactions such as bond adjustment, network formation, and activation mechanisms that determine the final material properties. In contrast, Fly Ash exhibited the least impact, indicating a secondary role compared to the other components. While Fly Ash is essential for the silica-alumina network, its influence on final strength is less pronounced than that of NaOH or Sludge, as evidenced by the lower SHAP values shown in Fig. 14(b). Based on these global findings, the optimization model focused on the dominant variables, NaOH, Sludge, Slag, and Gypsum for mixture fine-tuning.

### Optimization results

**Fig. 15:**
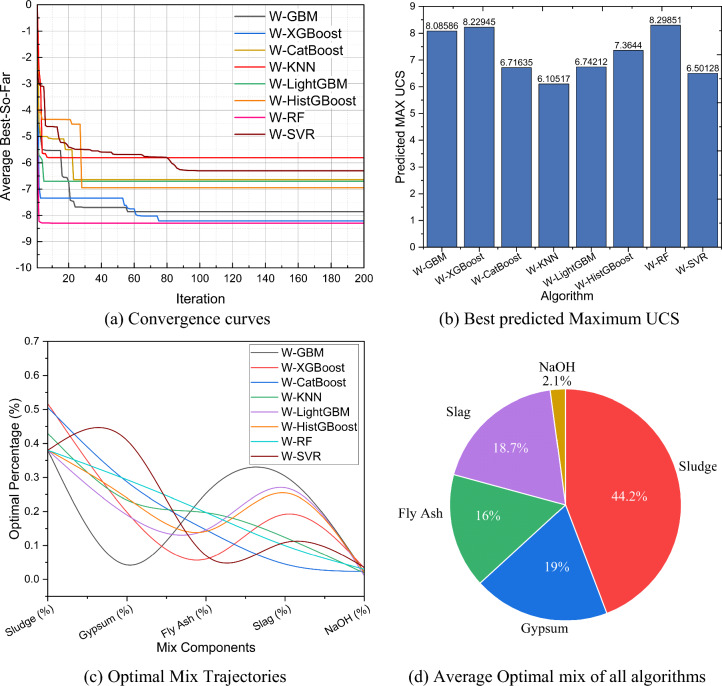
Multi-model optimization and performance analysis using the Hybrid ML-WOA approach.

For WOA-ML, the multi-model framework included eight algorithms: W-GBM, W-XGBoost, W-CatBoost, W-KNN, W-LightGBM, W-HistGBoost, W-RF and W-SVR. The optimization performance metrics are shown in Fig. 15, which includes convergence curves, bets predicted maximum UCS values, optimal mix trajectories, and the average optimal mix across all algorithms. Figure 15(a) shows the convergence curves, illustrating the evolution of the objective function value over 200 iterations for each hybrid model. Figure 15(a) shows the convergence curves of all hybrid models over 200 iterations. All models exhibited stable convergence, with objective values stabilizing after approximately 60–100 iterations, indicating satisfactory search efficiency of the WOA optimizer. GBM and XGBoost demonstrated the fastest convergence rates and reached near-optimal solutions at early iterations. RF and CatBoost also showed steady convergence but required more iterations. In contrast, KNN, LightGBM, and SVR converged more slowly and plateaued at comparatively higher objective values, reflecting weaker optimization performance. Figure 15(b) shows the best predicted UCS values obtained by each optimized model. RF achieved the highest predicted UCS (8.2985 MPa), followed by GBM (8.0586 MPa) and XGBoost (8.2295 MPa). CatBoost and HistGBoost produced moderate UCS values of 6.7164 MPa and 7.3644 MPa, respectively. LightGBM, KNN, and SVR showed relatively lower predictive performance, with maximum UCS values below 6.8 MPa. Figure 15(c) shows the optimal percentage trajectories for each component across the models. Figure 15(d) summarizes the average optimal mix in all ML-WOA models: sludge (44.2%), gypsum (19%), slag (18.7%), fly ash (16%), and NaOH (2.1%).

Table 11 summarizes the statistical performance of the WOA–ML models based on 30 independent runs, including mean UCS, standard deviation, and computational time. RF achieved the highest mean UCS (8.27 MPa), followed by XGBoost (8.22 MPa) and GBM (7.85 MPa), confirming their strong predictive capability. However, RF required the longest computational time (67.94 s), reflecting its high computational cost. In contrast, SVR exhibited the shortest runtime (2.51 s) but lower prediction accuracy. GBM offered a favorable trade-off between accuracy and efficiency, while KNN showed the weakest performance in both accuracy and stability. Table 12 presents pairwise statistical comparisons among the models. RF, GBM, and XGBoost achieved the highest number of wins with no losses. RF significantly outperformed CatBoost, KNN, LightGBM, HistGBoost, and SVR, while XGBoost and GBM also exceeded most competing models. CatBoost, LightGBM, and HistGBoost exhibited moderate performance, with multiple statistically insignificant differences, indicating comparable effectiveness. KNN recorded the most losses, confirming its limited suitability for UCS prediction. Overall, the analysis confirms that RF, XGBoost, and GBM are the most reliable models within the WOA–ML framework for optimizing sludge-based mixtures.

**Table 11:** Statistical results of different WOA-ML on UCS (30 independent runs).

Algorithm	Mean UCS (MPa)	Std UCS (MPa)	Time (s)	UCS rank
W-GBM	7.85415	0.68255	4.986	3
W-XGBoost	8.21625	0.70375	7.1272	2
W-CatBoost	6.63615	0.78425	9.2472	6
W-KNN	5.81325	0.91705	6.8151	8
W-LightGBM	6.6985	0.7671	8.7825	5
W-HistGBoost	6.9459	0.8194	38.7822	4
W-RF	8.26835	0.62815	67.9369	1
W-SVR	6.2525	0.7846	2.5091	7

**Table 12:** Pairwise statistical performance of WOA-ML models on UCS.

Comparison	Significant	Winner	p-value	Comparison		Significant	Winner	p-value
GBM vs XGBoost	=	Tie	0.2801	CatBoost vs LightGBM		=	Tie	0.7766
GBM vs CatBoost	+	GBM	0.0006	CatBoost vs HistGBoost		=	Tie	0.4645
GBM vs KNN	+	GBM	0.0000	CatBoost vs RF		-	RF	0.0000
GBM vs LightGBM	+	GBM	0.0099	CatBoost vs SVR		=	Tie	0.2801
GBM vs HistGBoost	+	GBM	0.0093	KNN vs LightGBM		-	LightGBM	0.0001
GBM vs RF	=	Tie	0.1241	KNN vs HistGBoost		-	HistGBoost	0.0002
GBM vs SVR	+	GBM	0.0001	KNN vs RF		-	RF	0.0000
XGBoost vs CatBoost	+	XGBoost	0.0006	KNN vs SVR		-	SVR	0.0016
XGBoost vs KNN	+	XGBoost	0.0000	LightGBM vs HitGBoost		=	Tie	0.4161
XGBoost vs LightGBM	+	XGBoost	0.0011	LightGBM vs RF		-	RF	0.0000
XGBoost vs HistGBoost	+	XGBoost	0.0001	LightGBM vs SVR		=	Tie	0.3085
XGBoost vs RF	=	Tie	0.4771	HistGBoost vs RF		-	RF	0.0000
XGBoost vs SVR	+	XGBoost	0.0000	HistGBoost vs SVR		+	HistGBoost	0.0197
CatBoost vs KNN	+	CatBoost	0.0003	RF vs SVR		+	RF	0.0000
Wins, Losses, and Ties for Each Algorithm								
Algorithms	GBM	XGBoost	RF	CatBoost	KNN	HistGBoost	LightGBM	SVR
+/-/=	5/0/2	5/0/2	5/02	1/1/5	0/6/1	2/1/4	0/3/4	1/3/3

+ / = / − indicate significantly better / no significant difference / significantly worse.

**Fig. 16:**
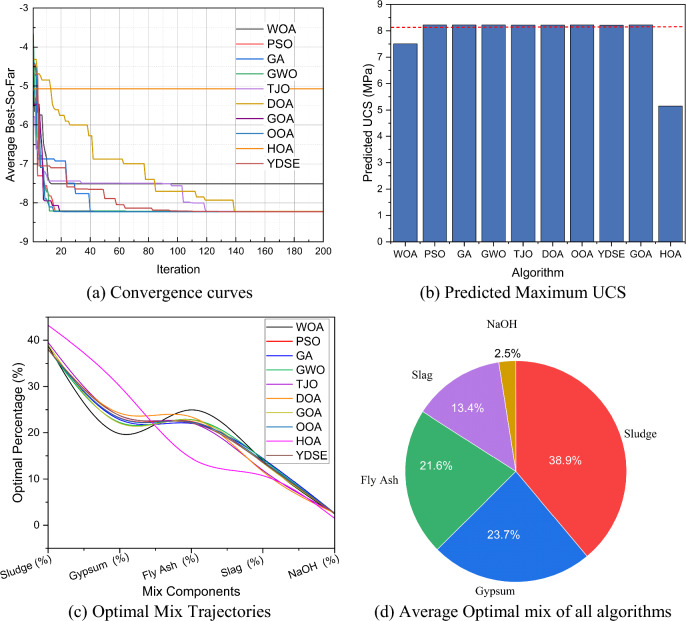
Multi-model optimization and performance analysis using various metaheuristic algorithms.

Regarding metaheuristic algorithms used to optimize the composition of slag–fly ash–gypsum blends for recycled sludge construction materials, the goal is to maximize UCS. The metaheuristic algorithms employed include WOA, PSO, GA, GWO, TJO, DOA, OOA, YDSE, GOA and HOA, each offering distinct advantages in navigating complex solution spaces and handling the nonlinear relationships inherent in the material mixtures. Figure 16(a) illustrates the convergence behavior of the metaheuristic algorithms over 200 iterations. Most population-based and swarm-based methods, including PSO, GA, GWO, TJO, DOA, GOA, OOA, and YDSE, exhibited rapid convergence and reached stable objective values within 50–100 iterations. These algorithms demonstrated efficient exploration and exploitation capabilities in the solution space. In contrast, WOA showed a more gradual convergence pattern and required a larger number of iterations to reach stability, reflecting its comparatively slower search mechanism. HOA also exhibited unstable convergence and premature stagnation, indicating limited optimization efficiency. Overall, swarm-based and evolutionary algorithms achieved faster and more stable convergence than WOA and HOA. Figure 16(b) presents the predicted maximum UCS values obtained by each algorithm. GWO, PSO, OOA, and GOA achieved the highest UCS values, approximately 8.22 MPa, followed closely by YDSE and TJO. GA also performed competitively, with a maximum UCS exceeding 8.08 MPa. WOA and HOA produced substantially lower UCS values, with HOA yielding the lowest result of 5.15 MPa. These results indicate that GWO, PSO, and OOA-based optimization strategies are more effective in identifying good solutions for sludge-based material mixtures. The optimal mixture trajectories obtained by each algorithm are shown in Fig. 16(c). Although variations exist among algorithms, all methods converged toward similar solution regions, particularly for sludge, gypsum, and fly ash contents, suggesting the existence of a stable optimal composition domain. Figure 16(d) summarizes the average optimal mixture proportions across all algorithms. The optimized composition consists of sludge (38.9%), gypsum (23.7%), fly ash (21.6%), slag (13.4%), and NaOH (2.5%). The dominant proportion of sludge and NaOH highlights their major contribution to UCS development.

Table 13 summarizes the statistical performance of the metaheuristic algorithms over 30 independent runs. GWO achieved the highest mean UCS (8.2267 MPa) with the lowest standard deviation (0.00138 MPa). PSO, OOA, and GOA also attained similarly high UCS values (≈8.225 MPa). Among these methods, OOA required the longest runtime and GOA exhibited high variability. YDSE and TJO demonstrated moderate performance, whereas GA and DOA showed reduced accuracy. WOA and HOA recorded the lowest UCS values, indicating poor reliability. Table 14 presents the results of pairwise statistical comparisons among the metaheuristic algorithms based on 30 independent runs. GWO and OOA achieved the highest number of wins (7 each), demonstrating excellent optimization performance. PSO, TJO, and GOA also showed competitive results, with eight wins each. In contrast, WOA and HOA recorded the highest number of losses, indicating weak competitiveness. GA, DOA, and YDSE exhibited moderate performance with mixed statistical outcomes. Overall, GWO exhibited the most consistent and reliable optimization performance.

**Table 13:** Statistical results of different MA on UCS (30 Independent Runs).

Algorithm	Mean UCS (MPa)	Std UCS (MPa)	Time (s)	UCS Rank
PSO	8.225109408	0.0054345	5.477817297	1
GA	8.08290472	0.274329751	5.746228933	7
GWO	8.226650225	0.00137755	8.308400154	4
TJO	8.14899322	0.194359131	7.012827873	6
OOA	8.226109408	0.00578954	11.75002408	1
YDSE	8.213324999	0.02769822	6.079662085	5
GOA	8.226109408	0.6478598	6.06479001	1
DOA	7.752386997	0.427637113	6.030442238	8
WOA	6.464645834	1.284270838	6.193442583	9
HOA	5.153661545	0.723184113	5.654686928	10

**Table 14:** Pairwise statistical performance of WOA-ML models on UCS.

**Comparison**	**Significant**	**Winner**	**p-value**	**Comparison**	**Significant**	Winner	p-value
WOA vs PSO	-	PSO	0.002	GA vs YDSE	=	Tie	0.2188
WOA vs GA	-	GA	0.002	GWO vs TJO	+	GWO	0.0039
WOA vs GWO	-	GWO	0.002	GWO vs DOA	+	GWO	0.002
WOA vs TJO	-	TJO	0.002	GWO vs GOA	=	Tie	1
WOA vs DOA	=	Tie	0.0645	GWO vs OOA	=	Tie	1
WOA vs GOA	-	GOA	0.002	GWO vs HOA	+	GWO	0.002
WOA vs OOA	-	OOA	0.002	GWO vs YDSE	+	GWO	0.0156
WOA vs HOA	+	WOA	0.0371	TJO vs DOA	+	TJO	0.0254
WOA vs YDSE	-	YDSE	0.002	TJO vs GOA	-	GOA	0.002
PSO vs GA	+	PSO	0.0039	TJO vs OOA	-	OOA	0.002
PSO vs GWO	=	Tie	1	TJO vs HOA	+	TJO	0.002
PSO vs TJO	+	PSO	0.002	TJO vs YDSE	=	Tie	0.625
PSO vs DOA	+	PSO	0.002	DOA vs GOA	-	GOA	0.002
PSO vs GOA	=	Tie	1	DOA vs OOA	-	OOA	0.002
PSO vs OOA	=	Tie	1	DOA vs HOA	+	DOA	0.002
PSO vs HOA	+	PSO	0.002	DOA vs YDSE	-	YDSE	0.0098
PSO vs YDSE	+	PSO	0.0078	GOA vs OOA	=	Tie	1
GA vs GWO	-	GWO	0.0039	GOA vs HOA	+	GOA	0.002
GA vs TJO	=	Tie	0.625	GOA vs YDSE	+	GOA	0.0078
GA vs DOA	=	Tie	0.1309	OOA vs HOA	+	OOA	0.002
GA vs GOA	-	GOA	0.0039	OOA vs YDSE	+	OOA	0.0078
GA vs OOA	-	OOA	0.0039	HOA vs YDSE	-	YDSE	0.002
GA vs HOA	+	GA	0.002				
Wins, Losses, and Ties for Each Algorithm							

Algorithms	PSO	GA	GWO	TJO	WOA	DOA	HOA	GOA	YDSE	OOA
+ /-/ =	6/4/0	4/6/0	7/3/0	6/4/0	2/8/0	3/7/0	2/8/0	6/4/10	6/4/0	7/3/0

+ / = / − indicate significantly better / no significant difference / significantly worse.

Table 15 summarizes the optimization results of the Hybrid ML-WOA approach and metaheuristic algorithms for determining the optimal mixture composition of modified sludge comprising gypsum, fly ash, slag, and NaOH, with the objective of maximizing UCS. Among the metaheuristic algorithms, PSO, GA, GOA, and OOA achieved highly competitive maximum UCS values, with GWO showing similarly strong performance, indicating effective exploration of the mixture space. TJO and DOA reached slightly lower maxima (~ 8.22 MPa), while HOA performed poorly, with a UCS of 5.15 MPa. Among the WOA–ML approaches, W-RF achieved the highest predicted UCS (8.2985 MPa), outperforming all metaheuristics. W-XGBoost also performed strongly (8.229 MPa), comparable to the best metaheuristics. W-GBM and W-HistGBoost achieved moderate UCS values (~ 7.36–8.08 MPa), whereas W-LightGBM, W-CatBoost, W-KNN, and W-SVR exhibited lower predictions (~ 6.1–6.74 MPa), indicating less effective guidance in mixture optimization. Overall, W-RF provided the highest UCS prediction and the most effective mixture optimization among all tested algorithms.

**Table 15:** Optimization results of different algorithms.

Algorithm	Composition (%)					Best UCS (MPa)
	Sludge	Gypsum	Fly Ash	Slag	NaOH	
WOA	38.8147	19.77052	24.94265	14.00035	2.471788	7.509392
PSO	38	23.2159	22.42716	13.7092	2.64774	8.226108
GA	38.09425	22.82417	22.02199	14.44593	2.613651	8.226107
GWO	38.12179	22.20948	22.83696	14.28112	2.550648	8.226109
TJO	39.55846	23.68957	22.14189	11.90089	2.709199	8.221518
DOA	38.10037	24.23234	23.41579	11.64331	2.608191	8.221428
GOA	39.02222	22.05711	22.76794	13.52004	2.63269	8.22611
OOA	38	23.13979	22.31534	13.90081	2.64406	8.22611
HOA	43.22797	31.64286	10.54614	13.10453	1.47850	5.15366
YDSE	38.00095	23.72021	22.13014	13.58377	2.56492	8.21332
W-GBM	38	4.25	25.33	30.38	2.04	8.08586
W-LightGBM	51.7	19.79	6.01	19.21	3.29	6.74212
W-XGBoost	50.59	28.71	14.61	4.5	2.34	8.22945
W-CatBoost	42.97	23.36	19.69	12.39	1.85	6.71635
W-KNN	38	18.98	14.61	27	1.05	6.10517
W-HistGBoost	38.038	23.989	14.16	25.5	1.92	7.3644
W-RF	38	29.3461	19.7165	9.9669	2.9706	8.29851
W-SVR	38	40.2523	7.2047	11.1529	3.516	6.50128

## Verification of optimized results

**Fig. 17:**
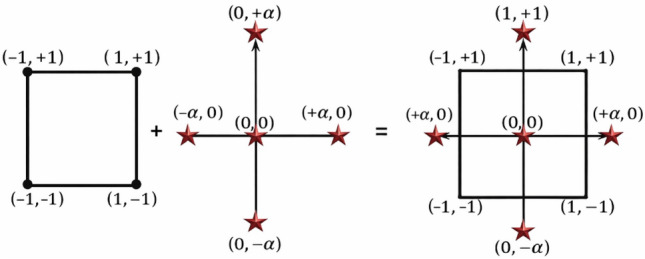
Central composite design (CCD) module.

The optimized results obtained from WOA-ML and MA were validated using Design-Expert® software to design the experimental plan. Response Surface Methodology (RSM) was employed to model and analyze the relationship between independent variables and the response (UCS). The procedure involved experimental design, data collection, model development, optimization, and validation. Among RSM designs, Central Composite Design (CCD) is widely used, consisting of factorial points (± 1), center points, and axial points (± α), as shown in Fig. 17.

A CCD with five factors (X₁ = Sludge, X₂ = Gypsum, X₃ = Fly Ash, X₄ = Slag, X₅ = NaOH) generated 149 experimental runs, including replicates at center points. The experimental data were analyzed using multiple regression with stepwise variable selection (α-to-enter = 0.15, α-to-remove = 0.15) to identify the most suitable model. Candidate terms included all linear terms (X₁–X₅), quadratic terms (X₁^2^–X₅^2^), and two-way interactions (X₁X₂–X₄X₅). Stepwise regression added terms sequentially based on statistical significance.

The response was approximated by a quadratic polynomial:

21 $$\mathrm{y}={\upbeta }_{0}+\sum_{\mathrm{i}=1}^{\mathrm{k}}{\upbeta }_{\mathrm{i}}{\mathrm{x}}_{\mathrm{i}}+\sum_{\mathrm{i}=1}^{\mathrm{k}}{\upbeta }_{\mathrm{ii}}{\mathrm{x}}_{\mathrm{i}}^{2}+\sum_{\mathrm{i}<\mathrm{j}}{\upbeta }_{\mathrm{ij}}{\mathrm{x}}_{\mathrm{i}}{\mathrm{x}}_{\mathrm{j}}+\upvarepsilon$$

where $\mathrm{y}$ denotes UCS, ${\mathrm{x}}_{\mathrm{i}}$(i = 1, …, k) are coded independent variables, ${\upbeta }_{0}$ is the intercept, ${\upbeta }_{\mathrm{i}}$ are linear coefficients, ${\upbeta }_{\mathrm{ii}}$ are quadratic coefficients, ${\upbeta }_{\mathrm{ij}}$ are interaction coefficients, and $\upvarepsilon$ is the random error term, assumed to be normally distributed with mean 0 and constant variance.

**Fig. 18:**
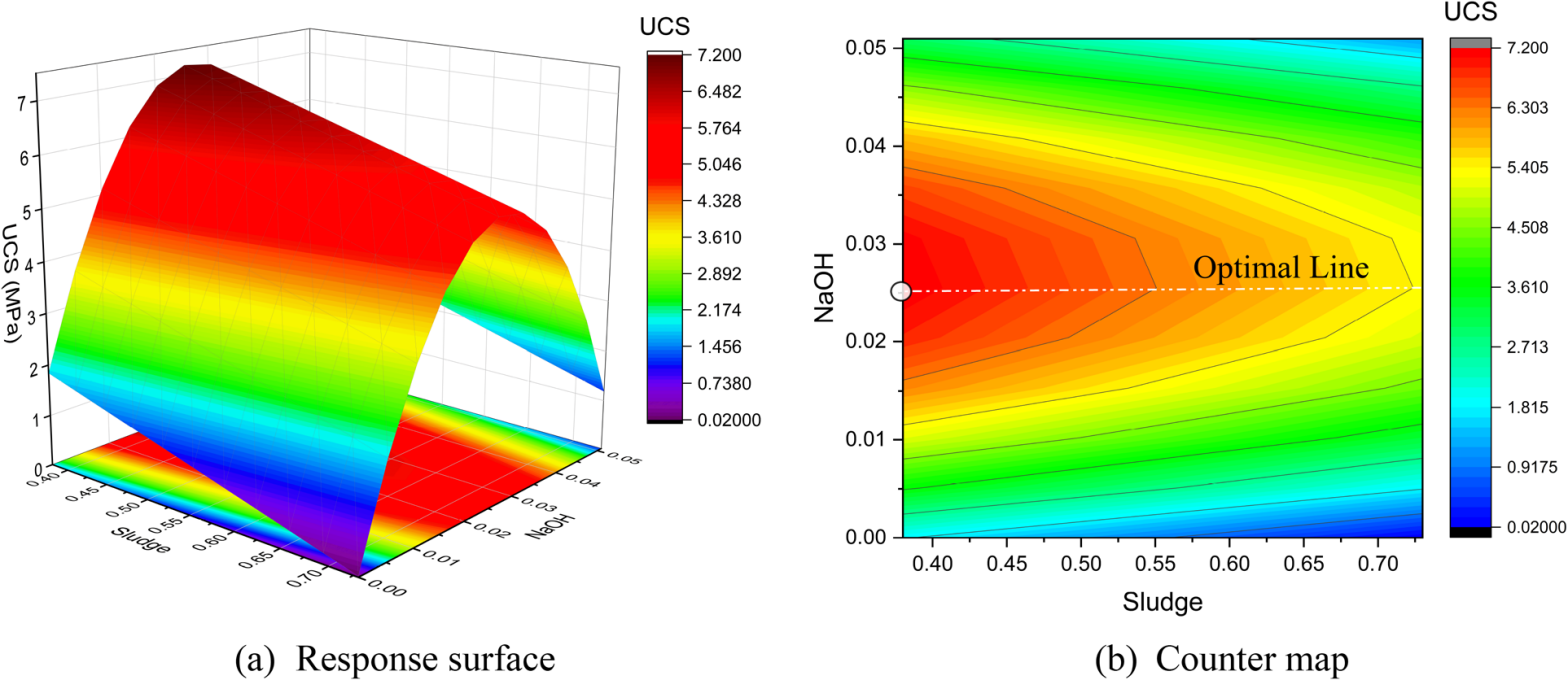
Response of sludge ratio and NaOH content on 28d UCS.

**Fig. 19:**
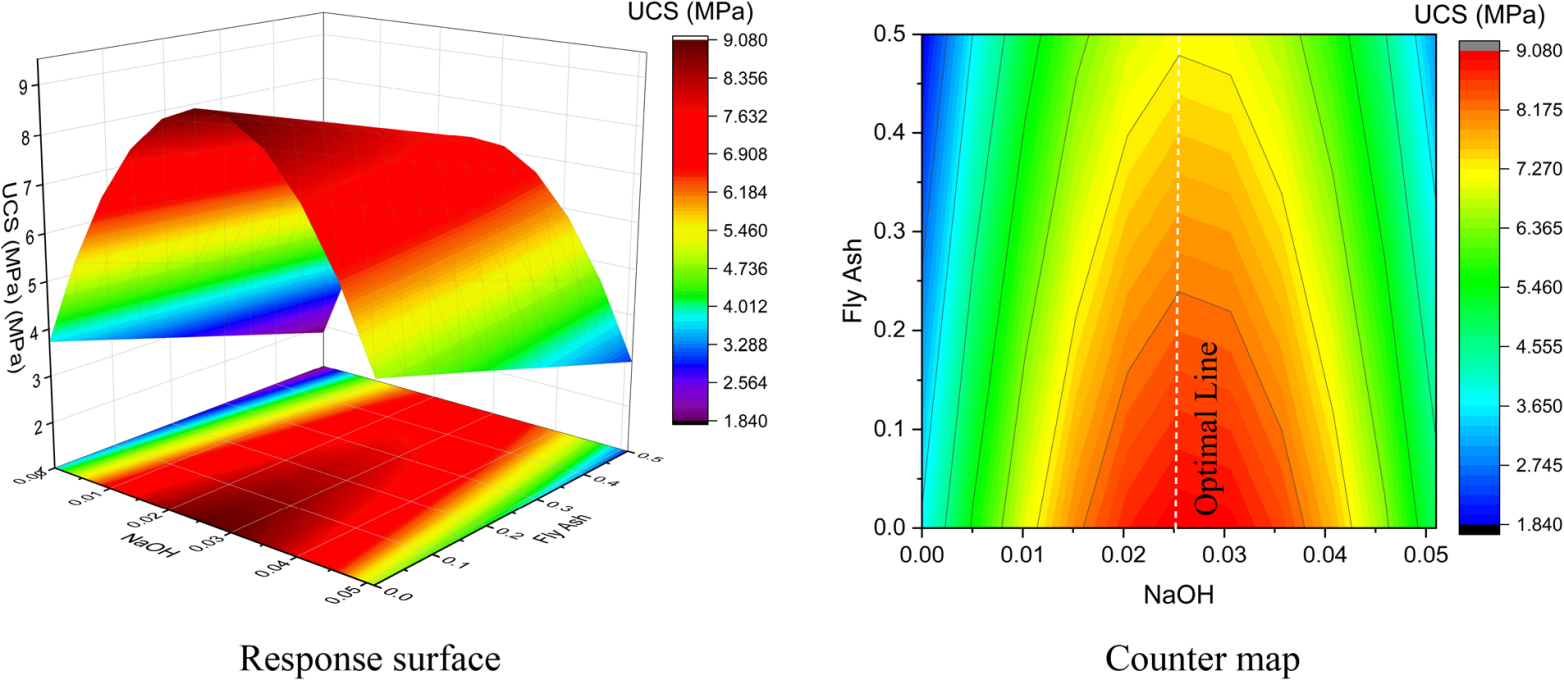
Response of NaOH ratio and fly ash content on 28d UCS.

Figures 18 and 19 present the response surface and contour plots of UCS, showing the effects of sludge ratio and NaOH content, and NaOH ratio and fly ash content, respectively, on 28-day UCS. A statistical model was developed to predict UCS, and the effects of three independent variables on the UCS of modified sludge were systematically evaluated. The model demonstrates a consistent correlation between UCS and the selected factors. An objective optimization technique was applied to maximize UCS while maintaining NaOH content at 0.5. The optimized results were validated experimentally, showing good agreement between predicted and observed UCS values. Consequently, RSM effectively predicted and optimized the UCS of modified sludge. The ANOVA model for UCS prediction is presented in Eq. (22).

22 $$\widehat{\mathrm{y}}=7.22523-0.90845{\mathrm{X}}_{1}-0.94510{\mathrm{X}}_{3}+0.58071{\mathrm{X}}_{5}-4.76656{\mathrm{X}}_{5}^{2}$$

where X₁, X₃, and X₅ represent sludge, fly ash, and NaOH content, respectively. The positive coefficient of X₅ and negative coefficient of X₅^2^ indicate a convex relationship, with UCS increasing to a maximum at an intermediate NaOH level (Figs. 18 and 19).

Table 16 presents the regression coefficients and statistical performance of the UCS model. All linear and quadratic terms were statistically significant (p < 0.001), and variance inflation factors below 3 indicated negligible multicollinearity. The model showed good accuracy (R^2^ = 0.955, Adj. R^2^ = 0.953) and satisfactory predictive capability (Pred. R^2^ = 0.921). Table 17 summarizes the ANOVA results. All model terms were significant (p < 0.001), with the quadratic term ${\mathrm{X}}_{5}^{2}$ exhibiting the highest F-value, indicating dominant nonlinear behavior. The low residual means square (0.35) reflected limited unexplained variability, confirming the validation of the proposed model.

**Table 16:** Regression coefficients, significance, and statistical performance of the UCS model.

Coded Coefficients								Statistics	
	Value	Std	95% LCL	95% UCL	t-Value	Prob >|t|	VIF	Metric	UCS
Intercept	7.22523	0.17702	6.87534	7.57512	40.81675	4.88486E-81		DF	144
X1	-0.90845	0.10755	-1.12103	-0.69586	-8.44651	2.96951E-14	2.01329	Root-MSE	0.59112
X3	-0.9451	0.09282	-1.12856	-0.76163	-10.18195	1.12205E-18	1.1454	R-Square	0.95483
X5	0.58071	0.12501	0.33362	0.82779	4.64545	7.58555E-6	2.10198	Adj. R^2^	0.95302
${\mathrm{x}}_{5}^{2}$	-4.76656	0.21173	-5.18507	-4.34806	-22.5121	5.12404E-49	2.26059	Pred. R^2^	0.92074

**Table 17:** ANOVA for UCS regression model.

Components	DF	Sum of Squares	Mean Square	F Value	Prob > F
X1	1	24.92932	24.92932	71.34356	2.96951E-14
X3	1	36.22579	36.22579	103.67218	1.12205E-18
X5	1	7.54068	7.54068	21.58017	7.58555E-6
${\mathrm{x}}_{5}^{2}$	1	177.08731	177.08731	506.79444	5.12404E-49
Error	144	50.31739	0.34943	-	-
Lack of fit	113	42.70625	0.37793	1.53931	0.08359
Pure Error	31	7.61114	0.24552	-	-
Total	148	772.14877			

**Fig. 20:**
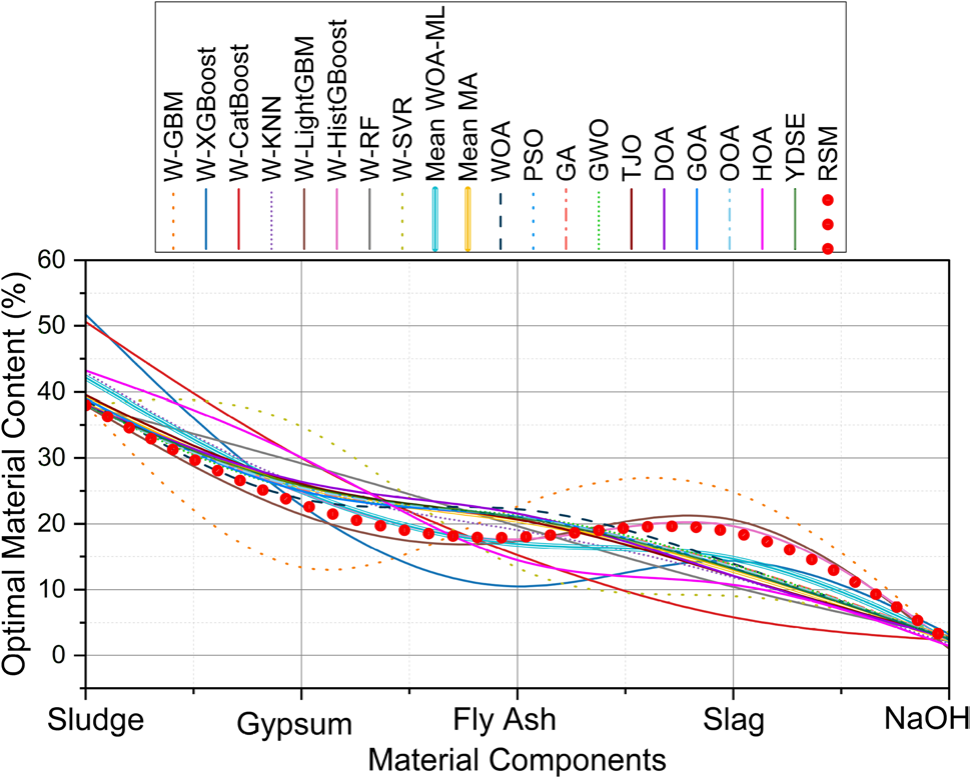
Comparison of RSM-optimized results with different WOA-ML and MA models.

Figure 20 shows the optimal results of RSM with material composition and denotes the optimal material content (%) to achieve the maximum predicted UCS. The optimized material contents obtained from various optimization frameworks, including WOA–ML and metaheuristic algorithms, were compared against the RSM-validated baseline, as illustrated in Fig. 20. Figure 20 indicate the RSM-optimized baseline. As observed, several machine learning–integrated models, particularly the Mean WOA–ML and Mean Metaheuristic Algorithm (MA), exhibit strong convergence with the RSM baseline, suggesting excellent predictive capability in determining optimal mix proportions.

## Conclusions

This study aims to combine machine learning and metaheuristic optimization to maximize the unconfined compressive strength of municipal sludge modified with slag, desulfurized gypsum, and fly ash. A total of 190 specimens were tested, and predictive models based on machine learning were coupled with the Whale Optimization Algorithm (WOA–ML), and metaheuristic algorithms were applied for comparison. Key conclusions are summarized as follows:

- The hybrid Whale Optimization Algorithm with machine learning (WOA–ML) models, particularly W-Random Forest (WOA–RF), achieved the highest predicted unconfined compressive strength of 8.2985 MPa. W-XGBoost and W-GBM also demonstrated strong performance, with predicted unconfined compressive strength values of 8.229 MPa and 8.085 MPa, respectively. The optimal mixture for maximum unconfined compressive strength consisted of sludge (44.2%), gypsum (19%), slag (18.7%), fly ash (16%), and NaOH (2.1%).
- Among the metaheuristic algorithms, Particle Swarm Optimization, Genetic Algorithm, Gazelle Optimization Algorithm, Octopus Optimization Algorithm, Hiking Optimization Algorithm, Double-Objective Algorithm, and Young’s Double-Slit Experiment optimizer all performed competitively. The Grey Wolf Optimizer achieved the highest unconfined compressive strength of 8.226 MPa, whereas the Hiking Optimization Algorithm was the lowest at 5.15 MPa, reflecting differences in exploration capabilities. On average, the optimal mix comprised 38.9% sludge, 23.7% gypsum, 21.6% fly ash, 13.4% slag, and 2.5% NaOH.
- Sensitivity analyses using SHAP and partial dependence confirmed that NaOH, sludge, slag, and gypsum were the most influential parameters on UCS, with NaOH being the most critical, validating the optimization results.
- Uncertainty quantification revealed that HistGBoost showed the lowest variance in predictions, whereas GBM, XGBoost, and CatBoost achieved a favorable balance between predictive accuracy and uncertainty. Bootstrap-based 95% prediction intervals confirmed reliable model calibration, with XGBoost providing the most consistent coverage.
- Response Surface Methodology (RSM) validation further confirmed that both WOA–ML and metaheuristic optimization approaches can reliably predict the optimal UCS of modified sludge.
- The hybrid WOA–ML framework offers several advantages, including accurate UCS prediction and efficient exploration of high-dimensional, nonlinear mixture spaces. Its main limitation is a slightly higher computational cost.
- The optimized results showed that sludge solidification can be used as a low-strength construction material.

## Data Availability

Some or all data, models, or code that support the findings of this study are available from the corresponding author upon reasonable request.

## Abbreviations

UCS: Unconfined compressive strength
AI: Artificial intelligence
ML: Machine learning
ANN: Artificial neural network
XGB: Extreme gradient boosting
GBM: Gradient boosting machine
KNN: K-nearest neighbors
HistGBoost: Histogram-based gradient boosting machine
CatBoost: Categorical boosting
UQ: Uncertainty quantification
KDE: Kernel density estimation
SHAP: SHapley additive explanations
WOA: Whale optimization algorithm
PSO: Particle swarm optimization
GA: Genetic algorithm
GWO: Grey wolf optimizer
TJO: Traffic jam optimization
DOA: Dream optimization algorithm
GOA: Gazelle optimization algorithm
HOA: Hiking optimization algorithm
OOA: Octopus optimization algorithm
YSDE: Young’s double-slit experiment optimizer
UCS: Unconfined compressive strength
R^2^: Coefficient of determination
RMSE: Root mean square error
MAE: Mean absolute error
sMAPE: Symmetric mean absolute percentage error
PBIAS: Percent bias
d: Index of agreement
NaoH: Sodium hydroxide
SiO₂: Silicon dioxide
Al₂O₃: Aluminum oxide
Fe₂O₃: Iron (III) oxide
CaO: Calcium oxide
MgO: Magnesium oxide
K₂O: Potassium oxide
Na₂O: Sodium oxide
*x*_1_: Sludge dosage (%)
*x*_2_: Gypsum dosage (%)
*x*_3_: Slag dosage (%)
*x*_4_: Fly ash dosage (%)
*x*_5_: NaOH dosage (%)
*Y*: Unconfined compressive strength
